# An Efficient Method for the Selective Syntheses of Sodium Telluride and Symmetrical Diorganyl Tellurides and the Investigation of Reaction Pathways

**DOI:** 10.3390/molecules29225398

**Published:** 2024-11-15

**Authors:** Chorong Kim, Yoo Jin Lim, Ye Eun Kim, Akula S. N. Murthy, Hyunsung Cho, Hyejeong Lee, Myung-Sook Park, Sang Hyup Lee

**Affiliations:** College of Pharmacy, Duksung Women’s University, 33, Samyangro 144-gil, Dobong-gu, Seoul 01369, Republic of Korea; giliiin0818@duksung.ac.kr (C.K.); esther1109@duksung.ac.kr (Y.J.L.); kimye92@duksung.ac.kr (Y.E.K.); surya.asnm@gmail.com (A.S.N.M.); sungcho2013@duksung.ac.kr (H.C.); hjeonglee@duksung.ac.kr (H.L.); mspark@duksung.ac.kr (M.-S.P.)

**Keywords:** tellurium, symmetrical diorganyl telluride, sodium telluride, sodium borohydride, reduction

## Abstract

Studies on organotellurium compounds have not been extensively conducted due to a lack of tolerable synthetic methods, difficult isolation processes, and their chemical instabilities. Overcoming these hurdles, we developed an efficient and mild method for the selective synthesis of symmetrical diorganyl tellurides **1**, a representative class of organotellurium compounds, using a proper reducing reagent. The reaction condition was optimized for the selective formation of **1** by forming the telluride dianion (Te^2−^) using a reducing reagent, sodium borohydride (NaBH_4_), and then followed by the addition of organyl halides. The optimized reaction condition was as follows: (1) Te (1.0 eq), NaBH_4_ (2.5 eq) in DMF for 1 h at 80 °C; (2) organyl halides (2.0 eq) for 3–5 h at 25–153 °C. Using this condition, 18 various diorganyl tellurides **1** were selectively and efficiently synthesized in reasonable yields (37–93%). The reaction pathways for the formation of diorganyl tellurides **1** were also investigated. Consequently, we established a practical and efficient method for the selective synthesis of diorganyl tellurides **1** as a representative class of organotellurium compounds.

## 1. Introduction

Tellurium (Te) is one of the chalcogen group of elements, together with oxygen (O), sulfur (S), and selenium (Se). Tellurium is often compared to sulfur or selenium based on its similar but different physicochemical properties. While sulfur and selenium compounds have shown excellent pharmacological effects [1], tellurium compounds have rarely been used in in vivo studies due to their toxicity, particularly their toxicity on the nervous system [2,3]. However, recent studies have reported that tellurium compounds have meaningful biological effects [4,5,6], and some of them revealed that organotellurium compounds show significantly higher efficacy compared to organoselenium compounds [7]. These are known to exhibit antitumor [5,6], antimicrobial [8,9,10], and antioxidant [11,12,13] activities. As a result, organotellurium compounds have been emerging as significant substances in the area of organic synthesis and medicinal chemistry over the years [4].

**Figure 1:**
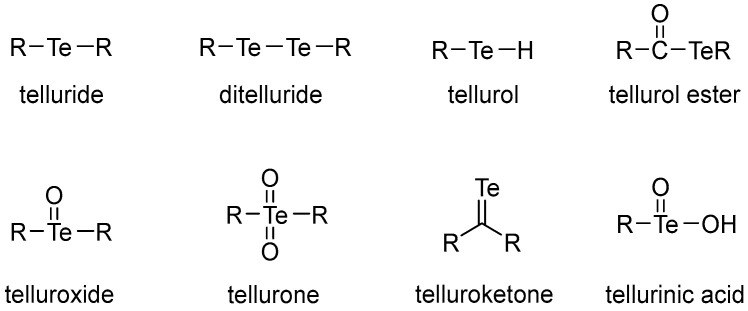
Types of organotellurium compounds.

The important classes of organotellurium compounds are presented in Figure 1. Among these, we are particularly interested in organyl telluride compounds based on their biological importance. Previously, we reported a study on the selective synthesis of diorganyl ditellurides (R-Te-Te-R) [14]. In addition, we were also interested in organoselenium compounds and reported studies on the selective synthesis of diorganyl diselenides (R-Se-Se-R) [15] and diorganyl selenides (R-Se-R) [16]. As an extension of our interest in chalcogen compounds, we aimed to develop efficient and tolerable methods for the selective synthesis of diorganyl tellurides **1**. As an example, we attempted to establish reaction conditions that facilitate the selective synthesis of diorganyl tellurides **1**. According to our preliminary studies, we chose NaBH_4_ as an appropriate reducing reagent to synthesize sodium telluride (Na_2_Te) because it can reduce elemental tellurium to Na_2_Te under milder conditions compared to other reducing reagents such as sodium (Na) or sodium hydride [17,18,19,20]. There have also been several studies on synthesizing organotellurium compounds using various reagents [21,22,23,24,25,26]. In this report, we describe a mild synthetic method for the selective synthesis of Na_2_Te and the corresponding diorganyl tellurides **1**.

## 2. Results and Discussion

### 2.1. Optimization for the Reaction Conditions

**Scheme 1:**
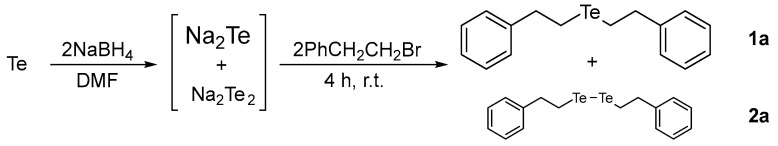
Formation of sodium telluride and bis(2-phenylethyl) telluride (**1a**).

To conduct selective syntheses of diorganyl tellurides **1**, we first investigated the reaction conditions for the selective formation of sodium telluride (Na_2_Te). Based on the previous procedures for ditellurides [14], we applied NaBH_4_ as a suitable reducing reagent for the formation of Na_2_Te with minor modifications. This choice was made to avoid the use of hazardous reducing reagents such as sodium (Na), sodium hydride, or hydrazine, after the consideration of previous studies and our preliminary experiments. As depicted in Scheme 1, we chose 2-phenethyl bromide (PhCH_2_CH_2_Br) as a template organyl halide to capture the Na_2_Te in situ.

One-pot reactions were performed using tellurium and NaBH_4_ in *N,N*-dimethylformamide (DMF) solvent. Given the importance of reducing reagents, we attempted to optimize the reaction conditions by adjusting the amount of NaBH_4_, temperature, and reaction time. The generated Na_2_Te was then directly reacted with PhCH_2_CH_2_Br to provide **1a** (Table 1). To ensure the reliability of the optimized conditions, we applied consistent conditions for the subsequent alkylation step (2.0 eq of PhCH_2_CH_2_Br, 25 °C, and 4 h).

In view of the theoretical amount, 2.0 eq of NaBH_4_ (compared to Te), we performed reactions using 2.0–3.0 eq of NaBH_4_ to ensure the full reduction of tellurium to telluride (oxidation state: −2), at temperatures ranging from 60 to 100 °C. As expected, we found that the amount of NaBH_4_ had a significant impact on the reaction. Considering the required theoretical amount (2.0 eq) of the reducing reagent, the use of 2.5 eq seemed excessive. However, we believed that the excess reducing reagents could be needed due to the additional consumption by oxygen or its incomplete working. Therefore, we concluded that 2.5 eq of reducing reagents would be a practically relevant amount for telluride formation at our reaction scale. Although the reactions at high temperatures (e.g., 100 °C) gave reasonable yields, the temperature was too high to be applied to the reactions with the organyl halide substrates with low boiling points.

Regarding the yields of the products and the selectivity of **1a** over **2a**, we have adopted the optimal conditions as follows: Te (1.0 eq), NaBH_4_ (2.5 eq), and a reaction time of 1 h at 80 °C; PhCH_2_CH_2_Br (2.0 eq) and a reaction time of 4 h at 25 °C.

### 2.2. Synthesis of Dioramic Tellurides **1**

Under the optimized conditions, we attempted to selectively synthesize diorganyl tellurides **1** by using various organyl halides (Table 2). We adopted primary, secondary, and tertiary alkyl halides, and aryl halides. At first, 2-phenethyl bromide afforded good yields (79%, Table 2, entry 1) as shown in Table 2. Other primary alkyl bromides such as *n*-butyl, *n*-pentyl, *n*-hexyl, *n*-heptyl, and *n*-octyl bromides also gave good to excellent yields (75–93%, entries 2 to 6). Then, secondary alkyl bromides gave moderate to good yields (40–76%, entries 7 to 11). Among these, *c*-hexyl bromide seemed to have lower reactivity than the others, requiring a longer reaction time (5 h) to provide **1j** (entry 10) in a relatively low yield (40%). Unfortunately, tertiary alkyl halides such as *t*-butyl bromide and even iodide did not give the product. We believed that tertiary alkyl halide could hardly react with Na_2_Te due to steric effects. When we used benzyl bromide, it did not provide good results either. To improve the results, we tried to elevate the reaction temperature (25–153 °C) and elongate reaction times (2–24 h). However, these reactions resulted in the gradual formation of a black solid during the reaction and the work-up process. It appeared that the generated product (dibenzyl telluride) might be decomposed and oxidized to elemental tellurium, which is also observed in our previous study [14]. This phenomenon is quite surprising and further investigation is underway.

When we conducted the reaction with aryl halides, we chose iodide instead of bromide due to iodide’s higher reactivity. Upon using unsubstituted phenyl iodide at 153 °C for 4 h, the resulting product was obtained in a good yield (67%, entry 12). We also conducted the reactions using phenyl iodides with electron-withdrawing groups (EWG), such as 3-(trifluoromethyl)phenyl, 4-(trifluoromethyl)phenyl, 4-nitrophenyl, and 4-cyanophenyl groups (entries 13 to 16). These reactions generally provided reasonable yields (37–45%). Additionally, 2-(trifluoromethyl)phenyl iodide, 2-cyanophenyl iodide, and 3-nitrophenyl iodide were also tested to examine the steric effect of the substituents on the phenyl ring, but they gave poor yields (<10%). We then conducted the reactions using phenyl iodides with electron-donating groups (EDG), such as 4-methylphenyl and 4-methoxyphenyl iodides (entries 17 and 18). 4-Methylphenyl iodide gave a relatively good yield (61%), while 4-methoxyphenyl iodide gave a low yield (43%). We believed that the aryl halides react with Na_2_Te through a nucleophilic aromatic substitution mechanism (S_N_Ar). Considering this mechanism, it was surprising that the reaction of phenyl iodide with EDG (e.g., 4-methylphenyl iodide) gave better yields than those of phenyl iodides with EWG. Notably, the results of aryl halides did not seem to consistently match the results expected by the S_N_Ar mechanism. The reactions of telluride species still seemed complex and unclear, and further studies are needed to clarify the reaction mechanism. Taken together, we have successfully synthesized eighteen diorganyl tellurides **1** with the minimal formation of diorganyl ditellurides **2**, among which four compounds (**1g**, **1h**, **1k**, and **1m**) are new.

### 2.3. Investigation of Reaction Pathways

**Scheme 2:**
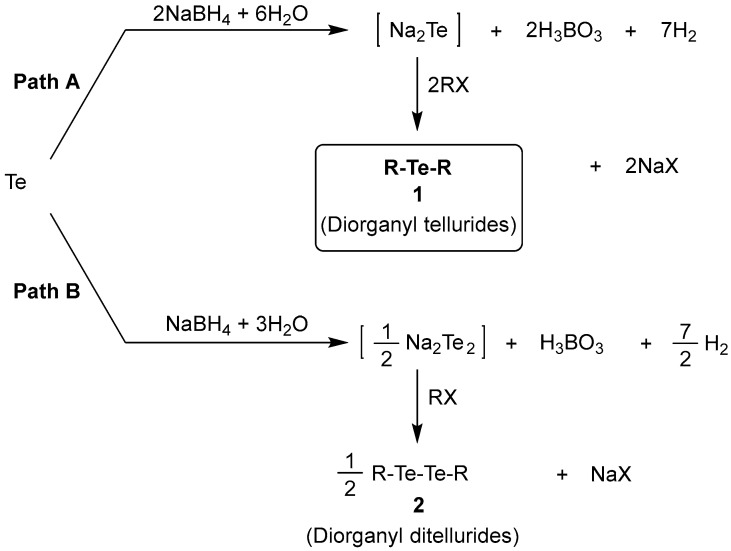
The proposed reaction pathways for diorgnyl tellurides **1** and diorganyl ditellurides **2**.

Based on these results, we have conducted studies on the reaction pathways. Here, we suggest the reaction pathways for diorganyl tellurides **1** and diorganyl ditellurides **2**, as shown in Scheme 2. First, for the synthesis of tellurides **1** (path A), NaBH_4_ (2.0 eq) reacts with Te (1.0 eq) in the presence of H_2_O, resulting in the formation of Na_2_Te (1.0 eq), H_3_BO_3_ (2.0 eq), and H_2_ gas (7.0 eq). Subsequently, the generated Na_2_Te (1.0 eq) is reacted with organyl halides (RX) (2.0 eq) to form the desired diorganyl tellurides **1**. According to the proposed reaction pathway, the theoretical amounts of reagents required for the formation of tellurides **1** (1.0 eq) are as follows: (1) Te (1.0 eq), NaBH_4_ (2.0 eq), and H_2_O (6.0 eq); (2) RX (2.0 eq). For the synthesis of ditellurides **2** (Path B), NaBH_4_ (1.0 eq) reacts with Te (1.0 eq) in the presence of H_2_O, giving Na_2_Te_2_ (0.5 eq), H_3_BO_3_ (1.0 eq), and H_2_ gas (3.5 eq). Subsequently, the generated Na_2_Te_2_ (0.5 eq) is reacted with organyl halides (RX) (1.0 eq) to form the diorganyl ditellurides **2**. Based on these proposed reaction pathways, the amount of reducing reagent (NaBH_4_) must be a significant factor in deciding the reaction pathway, Path A or Path B. Thus, according to the amount of reducing reagent, the equilibrium of the two pathways could shift between each other, resulting in the formation of a mixture of tellurides **1** and ditellurides **2**. A total of 2.0 eq of NaBH_4_ (compared to Te) reduces Te to Te^2−^ (oxidation state: −2), and 1.0 eq of NaBH_4_ reduces Te to Te_2_^2−^ (oxidation state: −1). In order to verify the proposed reaction pathways, we conducted the reactions by regulating the amount of NaBH_4_. For example, when we used 1.5 eq of NaBH_4_, the reactions followed not only Path A but also Path B [telluride **1** (Path A): ditellurides **2** (Path B)~1:0.8]. When we used 2.0 and 2.5 eq of NaBH_4_, the reactions selectively followed Path A. In reality, it was observed that a slightly higher quantity (2.5 eq) of NaBH_4_ than the theoretical amount (2.0 eq) gave the best result as shown in Table 1. In addition, it is believed that other reaction conditions such as reaction temperature and time should be carefully controlled to selectively synthesize product compounds **1** through Path A.

## 3. Experimental Procedure

### 3.1. General Methods

All reagents were purchased from commercial suppliers and used without further purification unless otherwise noted. Reaction progress was routinely monitored using thin-layer chromatography (TLC). Purification of the crude products was conducted via column chromatography, utilizing Merck silica gel (230–400 mesh). Melting and boiling points (uncorrected) were determined on round glass coverslips using a Fisher-Johns melting point apparatus (Waltham, MA, USA). FT-IR spectra were recorded using a PerkinElmer Spectrum GX spectrometer (Waltham, MA, USA) and frequencies (ν) are expressed in reciprocal centimeters (cm^−1^). ^1^H (400 MHz) and ^13^C (100 MHz) NMR spectra were recorded on an Agilent Technologies 400/54 spectrometer (Santa Clara, CA, USA), with chemical shifts (*δ*) reported relative to tetramethylsilane (TMS). Mass spectra were recorded in electron ionization (EI) mode. HPLC analyses were conducted with a Waters 1525 binary HPLC pump (Milford, MA, USA), a dual λ absorbance 2998 detector, and a COSMOSIL 5C_18_-AR-II Packed Column (Kyoto, Japan, 4.6 × 250 mm). Reaction products were analyzed under gradient conditions. Condition 1: from 50% A (aqueous) and 50% B (MeCN) in 3 min (isocratic) to 10% A and 90% B for 17 min (gradient), then 10% A and 90% B in 40 min (isocratic), and finally, from 50% A and 50% B over 5 min (gradient), then keep 50% A and 50% B for 5 min (isocratic). Condition 2: from 50% A (aqueous) and 50% B (MeCN) in 3 min (isocratic) to 10% A and 90% B in 15 min (gradient), then 10% A and 90% B for 25 min (isocratic), and finally, from 50% A and 50% B over 5 min (gradient). The flow rate was 1 mL/min with eluent monitoring at 254 nm. HPLC solvents were filtered (aqueous solution with Millipore HVLP, 0.45 mm; MeCN with Millipore HV, 0.45 mm, Burlington, MA, USA) and degassed before utilization.

### 3.2. General Procedure for the Synthesis of Diorganyl Tellurides **1**

Under a nitrogen atmosphere, Te (200 mg, 1.57 mmol, 1.0 eq) was added to a stirred solution of NaBH_4_ (149 mg, 3.92 mmol, 2.5 eq) in DMF solvent (2.4 mL). After stirring at 80 °C for 1 h, the reaction mixture turned a deep purple color (formation of Na_2_Te). Subsequently, the organyl halide was added, and the resulting mixture was stirred at 25–153 °C for 3–5 h (Table 2). After completion, the mixture was diluted with H_2_O (50 mL) and extracted twice with *n*-hexane or ethyl acetate (2 × 50 mL). The combined organic layers were washed with brine (50 mL), dried over anhydrous MgSO_4_, and concentrated under reduced pressure to give a sticky residue. This residue was purified via column chromatography, yielding diorganyl tellurides **1**. Spectra data of some compounds were in keeping with the literature information.

*Bis(2-phenylethyl) Telluride* (**1a**) [27]. The use of 2-phenethyl bromide (429 μL, 3.1 mmol, 2.0 eq) and a 4 h reaction time at 25 °C in the general procedure afforded the title compound **1a** (418 mg, 79%) as a bright yellow oil. Bp 140–142 °C; *R*_f_ 0.51 (1:2 Dichloromethane/*n*-hexane); HPLC t*_R_* 22.04 min (condition 1); λ_max_ = 348 nm; IR (ZnSe) 3024, 2925, 1493, 1148, 694 cm^−1^; ^1^H NMR (400 MHz, CDCl_3_): *δ* = 7.31–7.16 (m, 10 H, Ar), 3.05 (t, *J* = 8.0 Hz, 4 H, TeCH_2_), 2.83 (t, *J* = 8.0 Hz, 4 H, CH_2_); ^13^C NMR (100 MHz, CDCl_3_): *δ* = 142.73 (Ar), 128.50 (Ar), 128.12 (Ar), 126.30 (Ar), 38.74 (TeCH_2_), 3.47 (CH_2_); MS *m*/*z* 340 [M]^+^; HRMS (+EI) calcd for C_16_H_18_Te [M]^+^ 340.0471, found 340.0471.
*Di-n-butyl Telluride* (**1b**) [28]. The use of 1-bromobutane (339 μL, 3.1 mmol, 2.0 eq) and a 3 h reaction time at 25 °C in the general procedure afforded the title compound **1b** (292 mg, 77%) as a yellow oil. Bp 106–108 °C; *R*_f_ 0.44 (*n*-hexane); HPLC t*_R_* 24.67 min (condition 1); λ_max_ = 348, 240 nm; IR (ZnSe) 2956, 2924, 2870, 1459, 1160 cm^−1^; ^1^H NMR (400 MHz, CDCl_3_): *δ* = 2.63 (t, *J* = 7.5 Hz, 4 H, TeCH_2_), 1.72 (quintet, *J* = 7.4 Hz, 4 H, CH_2_), 1.38 (sextet, *J* = 7.4 Hz, 4 H, CH_2_), 0.92 (t, *J* = 7.5 Hz, 6 H, CH_3_); ^13^C NMR (100 MHz, CDCl_3_): *δ* = 34.42 (TeCH_2_), 25.13 (CH_2_), 13.43 (CH_2_), 2.37 (CH_2_); MS *m*/*z* 244 [M]^+^; HRMS (+EI) calcd for C_8_H_18_Te [M]^+^ 244.0471, found 244.0473.
*Di-n-pentyl Telluride* (**1c**) [29]. The use of 1-bromopentane (390 μL, 3.1 mmol, 2.0 eq) and a 3 h reaction time at 25 °C in the general procedure afforded the title compound **1c** (317 mg, 75%) as a bright yellow oil. Bp 122–124 °C; *R*_f_ 0.42 (*n*-hexane); HPLC t*_R_* 30.93 min (condition 1); λ_max_ = 345, 240 nm; IR (ZnSe) 2956, 2922, 2856, 1461, 1157 cm^−1^; ^1^H NMR (400 MHz, CDCl_3_): *δ* = 2.63 (t, *J* = 7.6 Hz, 4 H, TeCH_2_), 1.78–1.71 (m, 4 H, CH_2_), 1.39–1.31 (m, 8 H, CH_2_), 0.90 (t, *J* = 6.6 Hz, 6 H, CH_3_); ^13^C NMR (100 MHz, CDCl_3_): *δ* = 34.25 (TeCH_2_), 31.99 (CH_2_), 22.06 (CH_2_), 14.00 (CH_2_), 2.71 (CH_3_); MS *m*/*z* 272 [M]^+^; HRMS (+EI) calcd for C_10_H_22_Te [M]^+^ 272.0784, found 272.0781.
*Di-n-hexyl Telluride* (**1d**) [30]. The use of 1-bromohexane (457 μL, 3.1 mmol, 2.0 eq) and a 3 h reaction time at 25 °C in the general procedure afforded the title compound **1d** (380 mg, 81%) as a bright yellow oil. Bp 146–148 °C; *R*_f_ 0.47 (*n*-hexane); HPLC t*_R_* 41.21 min (condition 1); λ_max_ = 347, 240 nm; IR (ZnSe) 2956, 2922, 2853, 1461, 1155 cm^−1^; ^1^H NMR (400 MHz, CDCl_3_): *δ* = 2.63 (t, *J* = 7.6 Hz, 4 H, TeCH_2_), 1.77–1.70 (m, 4 H, CH_2_), 1.40–1.27 (m, 12 H, CH_2_), 0.89 (t, *J* = 6.5 Hz, 6 H, CH_3_); ^13^C NMR (100 MHz, CDCl_3_): *δ* = 32.27 (TeCH_2_), 31.75 (CH_2_), 31.20 (CH_2_), 22.58 (CH_2_), 14.07 (CH_2_), 2.76 (CH_3_); MS *m*/*z* 300 [M]^+^; HRMS (+EI) calcd for C_12_H_26_Te [M]^+^ 300.1097, found 300.1096.
*Di-n-heptyl Telluride* (**1e**) [31]. The use of 1-bromoheptane (493 μL, 3.1 mmol, 2.0 eq) and a 3 h reaction time at 25 °C in the general procedure afforded the title compound **1e** (470 mg, 92%) as an orange oil. Bp 168–170 °C; *R*_f_ 0.49 (*n*-hexane); HPLC t*_R_* 17.25 min (condition 2); λ_max_ = 347, 289, 240 nm; IR (ZnSe) 2956, 2921, 2852, 1459, 1154 cm^−1^; ^1^H NMR (400 MHz, CDCl_3_): *δ* = 2.62 (t, *J* = 7.6 Hz, 4 H, TeCH_2_), 1.77–1.70 (m, 4 H, CH_2_), 1.39–1.27 (m, 16 H, CH_2_), 0.88 (t, *J* = 6.9 Hz, 6 H, CH_3_); ^13^C NMR (100 MHz, CDCl_3_): *δ* = 32.31 (TeCH_2_), 32.04 (CH_2_), 31.99 (CH_2_), 28.67 (CH_2_), 22.65 (CH_2_), 14.09 (CH_2_), 2.77 (CH_3_); MS *m*/*z* 328 [M]^+^; HRMS (+EI) calcd for C_14_H_30_Te [M]^+^ 328.1410, found 328.1414.
*Di-n-octyl Telluride* (**1f**) [32]. The use of 1-bromooctane (541 μL, 3.1 mmol, 2.0 eq) and a 3 h reaction time at 25 °C in the general procedure afforded the title compound **1f** (515 mg, 93%) as a yellow oil. Bp 194–196 °C; *R*_f_ 0.49 (*n*-hexane); HPLC t*_R_* 29.68 min (condition 2); λ_max_ = 350, 240 nm; IR (ZnSe) 2956, 2921, 2852, 1460, 1153 cm^−1^; ^1^H NMR (400 MHz, CDCl_3_): *δ* = 2.62 (t, *J* = 7.6 Hz, 4H, TeCH_2_), 1.77–1.70 (m, 4 H, CH_2_), 1.39–1.26 (m, 20 H, CH_2_), 0.88 (t, *J* = 6.9 Hz, 6 H, CH_3_); ^13^C NMR (100 MHz, CDCl_3_): *δ* = 32.30 (TeCH_2_), 32.08 (CH_2_), 31.85 (CH_2_), 29.22 (CH_2_), 28.96 (CH_2_), 22.67 (CH_2_), 14.12 (CH_2_), 2.78 (CH_3_); MS *m*/*z* 356 [M]^+^; HRMS (+EI) calcd for C_16_H_34_Te [M]^+^ 356.1723, found 356.1719.
*Bis(3-pentyl) Telluride* (**1g**). The use of 3-bromopentane (390 μL, 3.1 mmol, 2.0 eq) and a 3 h reaction time at 25 °C in the general procedure afforded the title compound **1g** (259 mg, 61%) as an orange oil. Bp 91–92 °C (dec); *R*_f_ 0.45 (*n*-hexane); HPLC t*_R_* 28.80 min (condition 1); λ_max_ = 350, 293, 238 nm; IR (ZnSe) 2959, 2927, 2871, 2845, 1455, 1130 cm^−1^; ^1^H NMR (400 MHz, CDCl_3_): *δ* = 2.95 (quintet, *J* = 6.5 Hz, 2 H, TeCH), 1.82–1.71 (m, 8 H, CH_2_), 0.99 (t, *J* = 7.3 Hz, 12 H, CH_3_); ^13^C NMR (100 MHz, CDCl_3_): *δ* = 31.74 (TeCH), 30.54 (CH_2_), 13.91 (CH_3_); MS *m*/*z* 272 [M]^+^; HRMS (+EI) calcd for C_10_H_22_Te [M]^+^ 272.0784, found 272.0784.
*Bis(4-heptyl) Telluride* (**1h**). The use of 4-bromoheptane (493 μL, 3.1 mmol, 2.0 eq) and a 3 h reaction time at 25 °C in the general procedure afforded the title compound **1h** (389 mg, 76%) as an orange oil. Bp 100–102 °C (dec); *R*_f_ 0.45 (*n*-hexane); HPLC t*_R_* 12.40 min (condition 2); λ_max_ = 344 nm; IR (ZnSe) 2956, 2926, 2871, 1459, 1133 cm^−1^; ^1^H NMR (400 MHz, CDCl_3_): *δ* = 3.07 (quintet, *J* = 6.6 Hz, 2 H, TeCH), 1.75–1.63 (m, 8 H, CH_2_), 1.56–1.34 (m, 8 H, CH_2_), 0.92 (t, *J* = *7*.3 Hz, 12H, CH_3_); ^13^C NMR (100 MHz, CDCl_3_): *δ* = 40.47 (TeCH), 27.09 (CH_2_), 22.57 (CH_2_), 13.87 (CH_3_); MS *m*/*z* 328 [M]^+^; HRMS (+EI) calcd for C_14_H_30_Te [M]^+^ 328.1410, found 328.1409.
*Di-c-pentyl Telluride* (**1i**) [33]. The use of bromocyclopentane (318 μL, 3.1 mmol, 2.0 eq) and a 3 h reaction time at 25 °C in the general procedure afforded the title compound **1i** (262 mg, 63%) as a yellow oil. Bp 117–119 °C; *R*_f_ 0.39 (*n*-hexane); HPLC t*_R_* 8.18 min (condition 1); λ_max_ = 338 nm; IR (ZnSe) 2949, 2863, 1447, 1197 cm^−1^; ^1^H NMR (400 MHz, CDCl_3_): *δ* = 3.37 (quintet, *J* = 7.5 Hz, 2 H, TeCH), 2.18–2.12 (m, 4 H, CH_2_), 1.78–1.68 (m, 8 H, CH_2_), 1.61–1.54 (m, 4 H, CH_2_); ^13^C NMR (100 MHz, CDCl_3_): *δ* = 36.95 (TeCH), 25.52 (CH_2_), 18.39 (CH_2_); MS *m*/*z* 268 [M]^+^; HRMS (+EI) calcd for C_10_H_18_Te [M]^+^ 268.0471, found 268.0473.
*Di-c-hexyl Telluride* (**1j**) [19]. The use of bromocyclohexane (388 μL, 3.1 mmol, 2.0 eq) and a 5 h reaction time at 25 °C in the general procedure afforded the title compound **1j** (182 mg, 40%) as an orange oil. Bp 161–162 °C; *R*_f_ 0.38 (*n*-hexane); HPLC t*_R_* 9.94 min (condition 1); λ_max_ = 337 nm; IR (ZnSe) 2920, 2848, 1444, 1166 cm^−1^; ^1^H NMR (400 MHz, CDCl_3_): *δ* = 3.33–3.26 (m, 2 H, TeCH), 2.13–2.08 (m, 4 H, CH_2_), 1.78–1.59 (m, 10 H, CH_2_), 1.42–1.32 (m, 6 H, CH_2_); ^13^C NMR (100 MHz, CDCl_3_): *δ* = 37.25 (TeCH), 28.18 (CH_2_), 25.95 (CH_2_), 22.67 (CH_2_); MS *m*/*z* 296 [M]^+^; HRMS (+EI) calcd for C_12_H_22_Te [M]^+^ 296.0784, found 296.0785.
*Di-c-heptyl Telluride* (**1k**). The use of bromocycloheptane (431 μL, 3.1 mmol, 2.0 eq) and a 3 h reaction time at 25 °C in the general procedure afforded the title compound **1k** (249 mg, 49%) as an orange oil. Bp 156–158 °C; *R*_f_ 0.47 (*n*-hexane); HPLC t*_R_* 12.06 min (condition 1); λ_max_ = 347 nm; IR (ZnSe) 2917, 2850, 1454, 1182 cm^−1^; ^1^H NMR (400 MHz, CDCl_3_): *δ* = 3.44–3.38 (m, 2 H, TeCH), 2.20–2.14 (m, 4 H, CH_2_), 1.92–1.83 (m, 4 H, CH_2_), 1.68–1.43 (m, 16 H, CH_2_); ^13^C NMR (100 MHz, CDCl_3_): *δ* = 37.28 (TeCH), 28.20 (CH_2_), 25.97 (CH_2_), 22.68 (CH_2_); MS *m*/*z* 324 [M]^+^; HRMS (+EI) calcd for C_14_H_26_Te [M]^+^ 324.1097, found 324.1099.
*Diphenyl Telluride* (**1l**) [34]. The use of iodobenzene (350 μL, 3.1 mmol, 2.0 eq) and a 4 h reaction time at 153 °C in the general procedure afforded the title compound **1l** (295 mg, 67%) as a red oil. Bp 194–196 °C; *R*_f_ 0.50 (1:3 Dichloromethane/*n*-hexane); HPLC t*_R_* 19.36 min (condition 1); λ_max_ = 328, 283, 253 nm; IR (ZnSe) 3051, 1571, 1472, 1015, 725 cm^−1^; ^1^H NMR (400 MHz, CDCl_3_): *δ* = 7.70 (d, *J* = 7.2 Hz, 4 H, Ar), 7.30–7.18 (m, 6 H, Ar); ^13^C NMR (100 MHz, CDCl_3_): *δ* = 137.99 (Ar), 129.50 (Ar), 127.83 (Ar), 114.66 (Ar); MS *m*/*z* 284 [M]^+^; HRMS (+EI) calcd for C_12_H_10_Te [M]^+^ 283.9845, found 283.9842.
*Bis(3-(trifluoromethyl)phenyl) Telluride* (**1m**). The use of 3-iodobenzotrifluoride (452 μL, 3.1 mmol, 2.0 eq) and a 4 h reaction time at 153 °C in the general procedure afforded the title compound **1m** (251 mg, 38%) as a brown oil. Bp 165–167 °C; *R*_f_ 0.44 (1:10 Dichloromethane/*n*-hexane); HPLC t*_R_* 21.96 min (condition 1); λ_max_ = 290, 260 nm; IR (ZnSe) 2919, 1486, 1318, 1121, 791 cm^−1^; ^1^H NMR (400 MHz, CDCl_3_): *δ* = 7.96 (s, 2 H, Ar), 7.85 (d, *J* = 7.8 Hz, 2 H, Ar), 7.57 (d, *J* = 7.8 Hz, 2 H, Ar), 7.35 (t, *J* = 7.8 Hz, 2 H, Ar); ^13^C NMR (100 MHz, CDCl_3_): *δ* = 141.28 (Ar), 134.51 (q, ^3^*J*_CF_ = 3.8 Hz, Ar), 131.81 (q, ^2^*J*_CF_ = 32.4 Hz, Ar), 129.92 (Ar), 125.17 (q, ^3^*J*_CF_ = 3.8 Hz, Ar), 123.44 (q, ^1^*J*_CF_ = 273 Hz, CF_3_), 114.70 (Ar); MS *m*/*z* 420 [M]^+^; HRMS (+EI) calcd for C_14_H_8_F_6_Te [M]^+^ 419.9592, found 419.9595.
*Bis(4-(trifluoromethyl)phenyl) Telluride* (**1n**) [35]. The use of 4-iodobenzotrifluoride (462 μL, 3.1 mmol, 2.0 eq) and a 4 h reaction time at 153 °C in the general procedure afforded the title compound **1n** (295 mg, 45%) as an orange solid. Mp 52–54 °C (lit [35]. 52–53 °C); *R*_f_ 0.56 (1:10 Dichloromethane/*n*-hexane); HPLC t*_R_* 38.14 min (condition 1); λ_max_ = 225, 268, 299 nm; IR (ZnSe) 2930, 1596, 1393, 1320, 1161, 822 cm^−1^; ^1^H NMR (400 MHz, CDCl_3_): *δ* = 7.79 (d, *J* = 8.1 Hz, 4 H, Ar), 7.48 (d, *J* = 8.1 Hz, 4 H, Ar); ^13^C NMR (100 MHz, CDCl_3_): *δ* = 138.03 (Ar), 137.31 (Ar), 130.46 (q, ^2^*J*_CF_ = 32.8 Hz, Ar), 126.28 (q, ^3^*J*_CF_ = 3.8 Hz, Ar), 123.94 (q, ^1^*J*_CF_ = 272 Hz, CF_3_); MS *m*/*z* 420 [M]^+^; HRMS (+EI) calcd for C_14_H_8_F_6_Te [M]^+^ 419.9592, found 419.9594.
*Bis(4-nitrophenyl) Telluride* (**1o**) [36]. The use of 4-iodonitrobenzene (782 mg, 3.1 mmol, 2.0 eq) and a 4 h reaction time at 153 °C in the general procedure afforded the title compound **1o** (235 mg, 40%) as an orange solid. Mp 170–172 °C (lit [36]. 170–172 °C); *R*_f_ 0.18 (1:1 Dichloromethane/*n*-hexane); HPLC t*_R_* 12.74 min (condition 1); λ_max_ = 318, 238 nm; IR (ZnSe) 3056, 1590, 1499, 1335, 836 cm^−1^; ^1^H NMR (400 MHz, CDCl_3_): *δ* = 8.08 (d, *J* = 8.3 Hz, 4 H, Ar), 7.83 (d, *J* = 8.2 Hz, 4 H, Ar); ^13^C NMR (100 MHz, CDCl_3_): *δ* = 138.25 (Ar), 137.32 (Ar), 124.40 (Ar), 123.94 (Ar); MS *m*/*z* 374 [M]^+^; HRMS (+EI) calcd for C_12_H_8_N_2_O_4_Te [M]^+^ 373.9546, found 373.9547.
*Bis(4-cyanophenyl) Telluride* (**1p**) [37]. The use of 4-iodobenzonitrle (719 mg, 3.1 mmol, 2.0 eq) and a 4 h reaction time at 153 °C in the general procedure afforded the title compound **1p** (192 mg, 37%) as an orange solid. Mp 140–141 °C (lit [37]. 141 °C); *R*_f_ 0.28 (1:1 Dichloromethane/*n*-hexane); HPLC t*_R_* 12.83 min (condition 1); λ_max_ = 318, 238 nm; IR (ZnSe) 3081, 2222, 1580, 1477, 812 cm^−1^; ^1^H NMR (400 MHz, CDCl_3_): *δ* = 7.76 (d, *J* = 7.5 Hz, 4 H, Ar), 7.50 (d, *J* = 7.5 Hz, 4 H, Ar); ^13^C NMR (100 MHz, CDCl_3_): *δ* = 138.13 (Ar), 137.35 (Ar), 132.81 (Ar), 132.44 (Ar), 118.27 (CN); MS *m*/*z* 334 [M]^+^; HRMS (+EI) calcd for C_14_H_8_N_2_Te [M]^+^ 333.9750, found 333.9749.
*Bis(4-methylphenyl) Telluride* (**1q**) [36]. The use of 4-iodotoluene (685 mg, 3.1 mmol, 2.0 eq) and a 4 h reaction time at 153 °C in the general procedure afforded the title compound **1q** (294 mg, 61%) as an orange solid. Mp 61–62 °C (lit [36]. 67 °C); *R*_f_ 0.30 (1:20 Dichloromethane/*n*-hexane); HPLC t*_R_* 23.24 min (condition 1); λ_max_ = 328, 281, 234, 211 nm; IR (ZnSe) 3019, 1484, 1008, 797 cm^−1^; ^1^H NMR (400 MHz, CDCl_3_): *δ* = 7.58 (d, *J* = 7.8 Hz, 4 H, Ar), 7.02 (d, *J* = 8.0 Hz, 4 H, Ar), 2.32 (s, 6 H, CH_3_); ^13^C NMR (100 MHz, CDCl_3_): *δ* = 138.08 (Ar), 137.75 (Ar), 130.37 (Ar), 130.13 (Ar), 21.20 (CH_3_); MS *m*/*z* 312 [M]^+^; HRMS (+EI) calcd for C_14_H_14_Te [M]^+^ 312.0158, found 312.0161.
*Bis(4-methoxyphenyl) Telluride* (**1r**) [36]. The use of 4-iodoanisole (735 mg, 3.1 mmol, 2.0 eq) and a 4 h reaction time at 153 °C in the general procedure afforded the title compound **1r** (231 mg, 43%) as a brown solid. Mp 51–53 °C (lit [36]. 53–54 °C); *R*_f_ 0.40 (1:1 Dichloromethane/*n*-hexane); HPLC t*_R_* 18.24 min (condition 1); λ_max_ = 245 nm; IR (ZnSe) 3006, 2942, 2840, 1487, 1280, 1026, 832 cm^−1^; ^1^H NMR (400 MHz, CDCl_3_): *δ* = 7.62 (d, *J* = 8.0 Hz, 4 H, Ar), 6.76 (d, *J* = 8.0 Hz, 4 H, Ar), 3.78 (s, 6 H, CH_3_); ^13^C NMR (100 MHz, CDCl_3_): *δ* = 159.68 (Ar), 139.72 (Ar), 115.39 (Ar), 104.30 (Ar), 55.16 (CH_3_); MS *m*/*z* 344 [M]^+^; HRMS (+EI) calcd for C_14_H_14_O_2_Te [M]^+^ 344.0056, found 344.0058.

## 4. Conclusions

We report an efficient and tolerable method for the selective synthesis of Na_2_Te and the corresponding diorganyl tellurides **1** instead of diorganyl ditellurides **2**. We aimed to optimize the reactions using NaBH_4_ under mild and convenient conditions, avoiding the use of hazardous reagents such as sodium (Na), sodium hydride, or hydrazine. The optimal conditions were as follows: (1) Te (1.0 eq), NaBH_4_ (2.5 eq) in DMF for 1 h at 80 °C; (2) organyl halides (2.0 eq) for 3–5 h at 25–153 °C. Using these optimized conditions, we successfully synthesized eighteen diorganyl tellurides **1**, among which four compounds (**1g**, **1h**, **1k**, and **1m**) are new. The primary alkyl bromides gave **1a**–**1f** in good to excellent yields (75–93%), while the secondary alkyl bromides provided **1g**–**1k** in modest to good yields (40–76%). The aryl iodides gave **1l**–**1r** in modest to good yields (37–67%). Additionally, we conducted mechanistic studies to understand the reaction pathways for the formation of Na_2_Te and diorganyl tellurides **1**. Consequently, we have achieved a systematic study on the selective syntheses of Na_2_Te and the corresponding diorganyl telluride compounds **1** using NaBH_4_ as a convenient reducing reagent.

## Figures and Tables

**Table 1:**
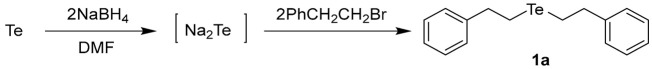
Optimized reaction method using the reducing reagent NaBH_4_^a^.

Entry	Na_2_Te Formation ^a^			Yields (%) (1a:2a) ^b^	Yields (%) (1a) ^c^
	NaBH_4_ (eq)	Temp (°C)	Time (h)		
1	2.0	60	1.0	50 (8.0:1)	44
2			2.0	49 (6.4:1)	42
3		80	1.0	73 (23:1)	70
4			2.0	80 (12:1)	74
5		100	0.5	72 (17:1)	68
6			1.0	84 (8.3:1)	75
7	2.5	60	1.0	67 (15:1)	63
8			2.0	80 (21:1)	76
9		80	0.5	64 (1.0:0)	64
10			1.0	79 (1.0:0)	79
11			2.0	61 (7.0:1)	53
12		100	0.5	65 (2.6:1)	47
13			1.0	69 (15:1)	65
14	3.0	60	1.0	67 (1.0:0)	67
15			2.0	73 (42:1)	71
16		80	0.5	62 (1.0:0)	62
17			1.0	63 (32:1)	61
18		100	0.5	68 (10:1)	62
19			1.0	74 (6.3:1)	64

^a^ All reactions were run on a 1.57 mmol scale of Te (1.0 eq, [c] = 654 mM) in DMF (2.4 mL) for the formation of Na_2_Te; PhCH_2_CH_2_Br (2.0 eq) at 25 °C for 4 h for the formation of **1a**; ^b^ The ratio was determined by ^1^H-NMR; ^c^ Isolated yields.

**Table 2:**
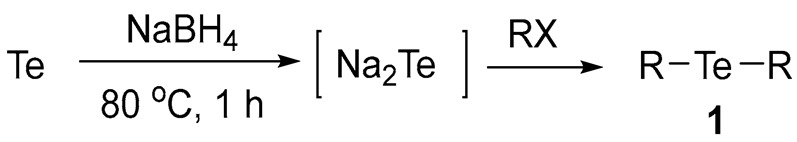
Synthesis of diorganyl tellurides **1** ^a^.

Entry	Reaction with RX				Product	Yields (%) ^b^
	RX	RX (eq)	Time (h)	Temp (°C)		
1	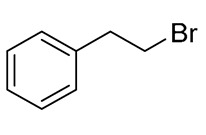	2.0	4	25	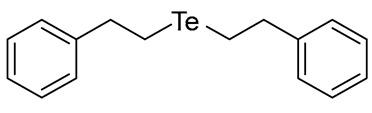 **1a**	79
2	*n*-BuBr	2.0	3	25	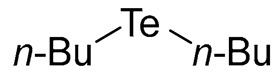 **1b**	77
3	*n*-PenBr	2.0	3	25	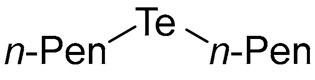 **1c**	75
4	*n*-HexBr	2.0	3	25	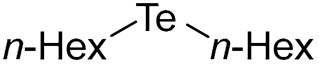 **1d**	81
5	*n*-HepBr	2.0	3	25	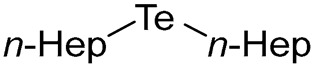 **1e**	92
6	*n*-OctBr	2.0	3	25	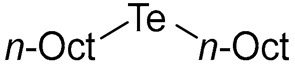 **1f**	93
7	3-Br-Pen	2.0	3	25	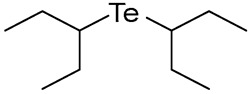 **1g**	61
8	4-Br-Hep	2.0	3	25	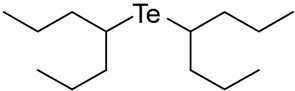 **1h**	76
9	*c*-PenBr	2.0	3	25	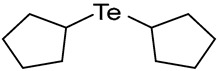 **1i**	63
10	*c*-HexBr	2.0	5	25	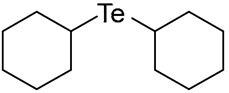 **1j**	40
11	*c*-HepBr	2.0	3	25	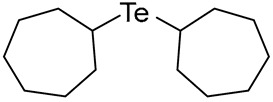 **1k**	49
12	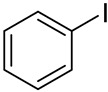	2.0	4	153	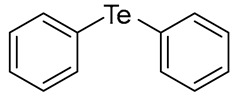 **1l**	67
13	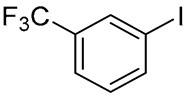	2.0	4	153	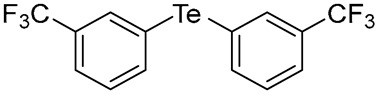 **1m**	38
14	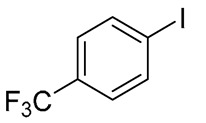	2.0	4	153	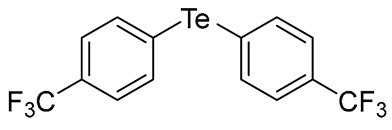 **1n**	45
15	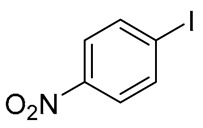	2.0	4	153	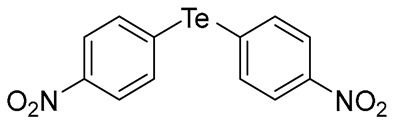 **1o**	40
16	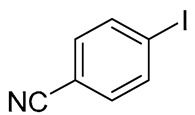	2.0	4	153	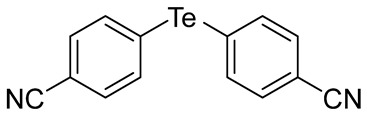 **1p**	37
17	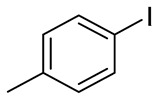	2.0	4	153	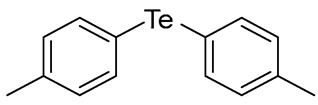 **1q**	61
18	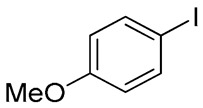	2.0	4	153	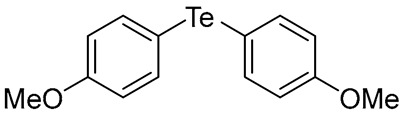 **1r**	43

^a^ All reactions were run on a 1.6 mmol scale of Te (1.0 eq, [c] = 650 mM) in DMF (2.4 mL) and NaBH_4_ (2.5 eq) for 1 h at 80 °C for the formation of Na_2_Te; ^b^ Isolated yields.

## Data Availability

The data presented in this study are available in the article and Supplementary Materials.

## Supplementary Materials

The following supporting information can be downloaded at: https://www.mdpi.com/article/10.3390/molecules29225398/s1. The charts for ^1^H- and ^13^C-NMR spectroscopies.
