# Supplementary material for: An Efficient Method for the Selective Syntheses of Sodium Telluride and Symmetrical Diorganyl Tellurides and the Investigation of Reaction Pathways
Source: Molecules. 2024 Nov 15;29(22):5398. doi: 10.3390/molecules29225398 (PMC11597708; doi:10.3390/molecules29225398)
Supplement: Supplementary file 1 [file molecules-29-05398-s001.zip › molecules-3286911-supplementary.pdf]

Supplementary data

for

An Efficient Method for the Selective Syntheses of Sodium Telluride and Symmetrical Diorganyl Tellurides and the Investigation of Reaction Pathways

Chorong Kim, Yoo Jin Lim, Ye Eun Kim, Akula S. N. Murthy, Hyunsung Cho, Hyejeong Lee, Myung-Sook Park, and Sang Hyup Lee*

College of Pharmacy and Innovative Drug Center, Duksung Women's University, Seoul 01369, Republic of Korea,

*sanghyup@duksung.ac.kr

^{1}H and ^{13}C NMR spectrum of compound **1a** S2
^{1}H and ^{13}C NMR spectrum of compound **1b** S3
^{1}H and ^{13}C NMR spectrum of compound **1c** S4
^{1}H and ^{13}C NMR spectrum of compound **1d** S5
^{1}H and ^{13}C NMR spectrum of compound **1e** S6
^{1}H and ^{13}C NMR spectrum of compound **1f** S7
^{1}H and ^{13}C NMR spectrum of compound **1g** S8
^{1}H and ^{13}C NMR spectrum of compound **1h** S9
^{1}H and ^{13}C NMR spectrum of compound **1i** S10
^{1}H and ^{13}C NMR spectrum of compound **1j** S11
^{1}H and ^{13}C NMR spectrum of compound **1k** S12
^{1}H and ^{13}C NMR spectrum of compound **1l** S13
^{1}H and ^{13}C NMR spectrum of compound **1m** S14
^{1}H and ^{13}C NMR spectrum of compound **1n** S15
^{1}H and ^{13}C NMR spectrum of compound **1o** S16
^{1}H and ^{13}C NMR spectrum of compound **1p** S17
^{1}H and ^{13}C NMR spectrum of compound **1q** S18
^{1}H and ^{13}C NMR spectrum of compound **1r** S19

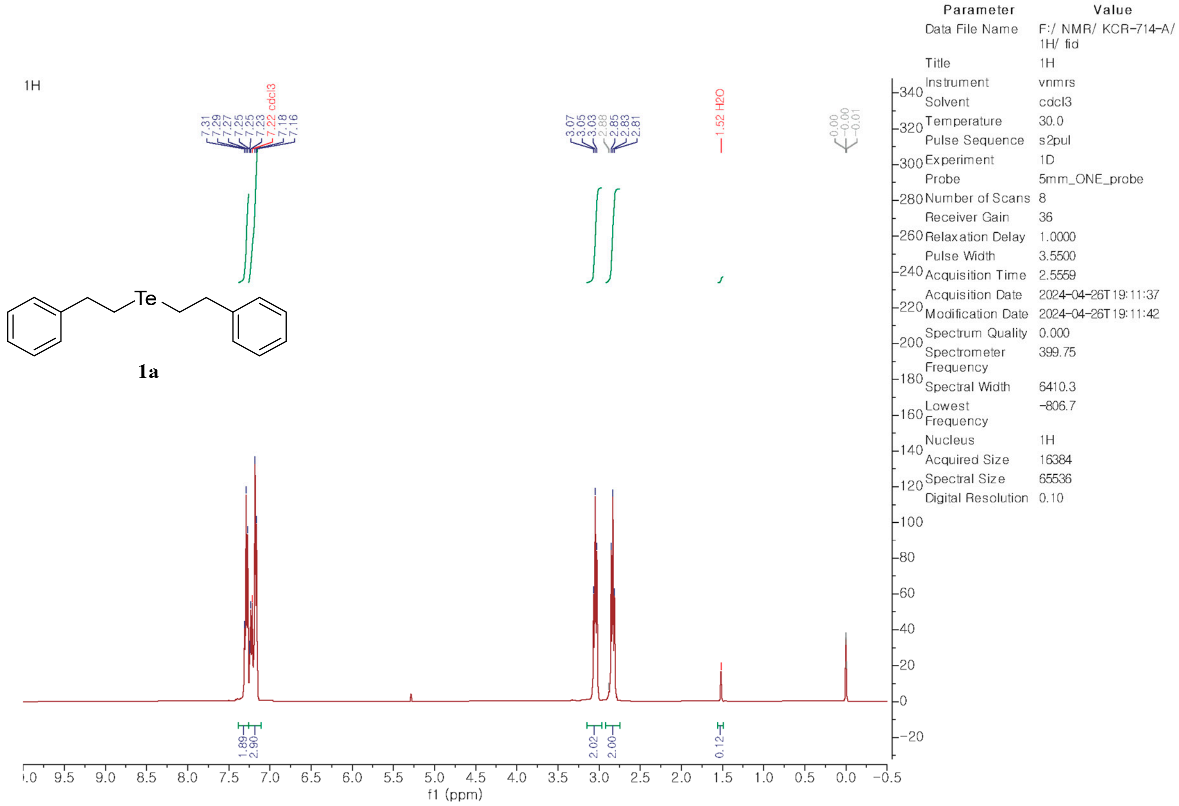

^{1}H NMR spectrum (400 MHz, $CDCl_3$) of compound 1a

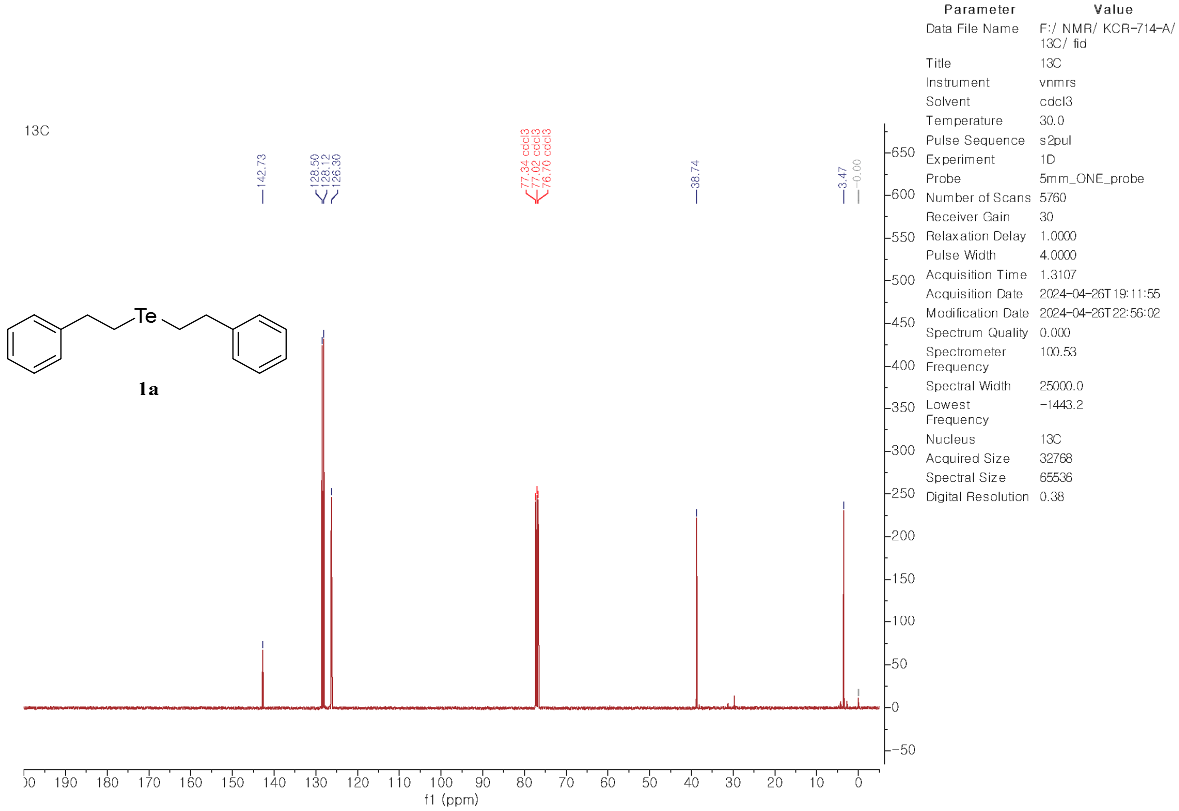

^{13}C NMR spectrum (100 MHz, $CDCl_3$) of compound 1a

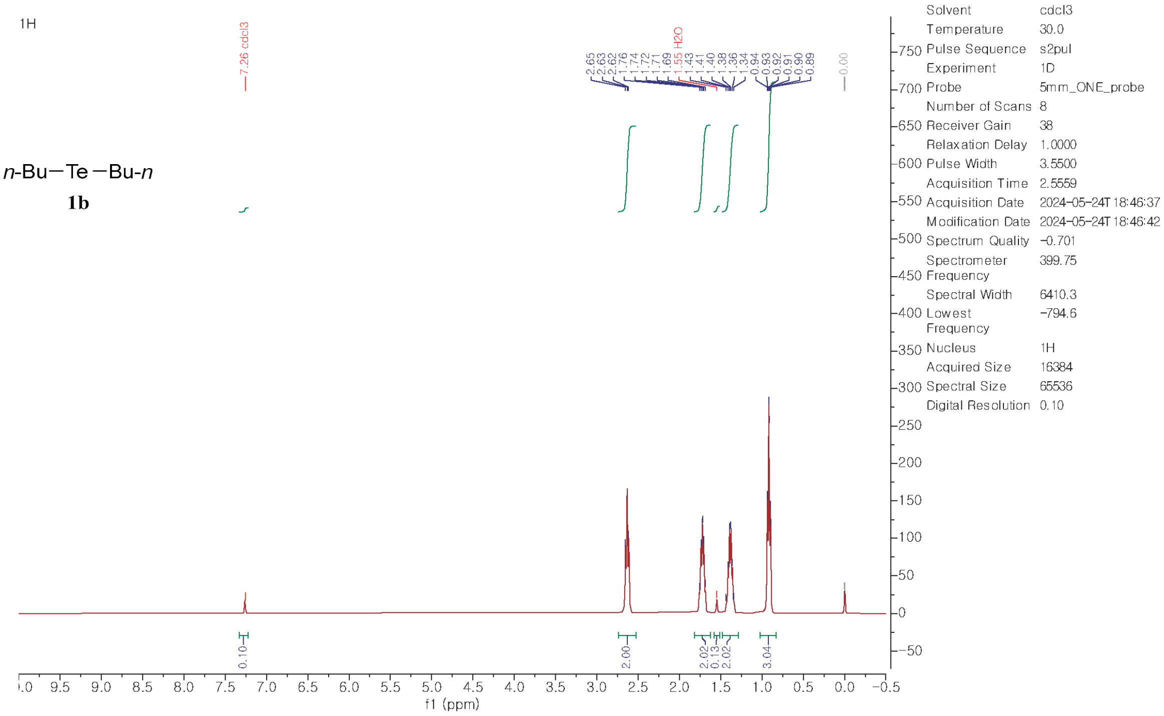

Parameter	Value
Data File Name	F:/ NMR/ KCR-732-A/ 1H/ fid
Title	1H
Instrument	vnmrs
Solvent	cdcl3
Temperature	30.0
Pulse Sequence	s2pul
Experiment	1D
Probe	5mm_ONE_probe
Number of Scans	8
Receiver Gain	38
Relaxation Delay	1.0000
Pulse Width	3.5500
Acquisition Time	2.5559
Acquisition Date	2024-05-24T18:46:37
Modification Date	2024-05-24T18:46:42
Spectrum Quality	-0.701
Spectrometer Frequency	399.75
Spectral Width	6410.3
Lowest Frequency	-794.6
Nucleus	1H
Acquired Size	16384
Spectral Size	65536
Digital Resolution	0.10

^{1}H NMR spectrum (400 MHz, $CDCl_3$) of compound 1b

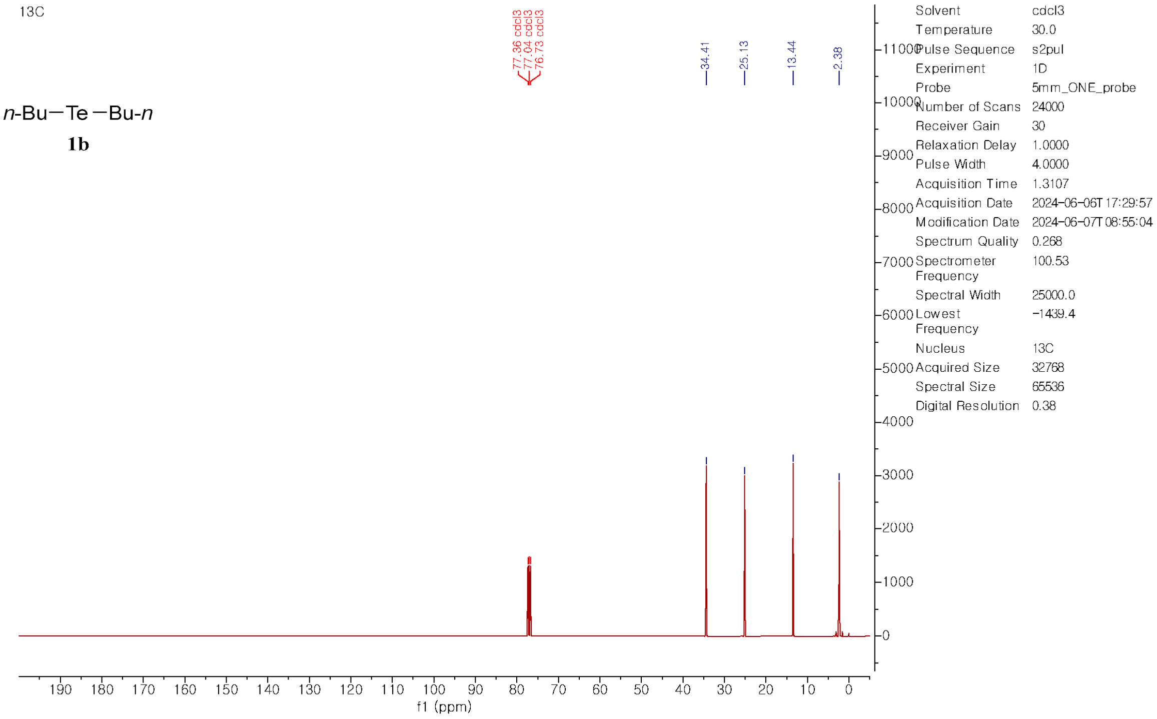

Parameter	Value
Data File Name	F:/ NMR/ KCR-732-A/ 13C/ fid
Title	13C
Instrument	vnmrs
Solvent	cdcl3
Temperature	30.0
Pulse Sequence	s2pul
Experiment	1D
Probe	5mm_ONE_probe
Number of Scans	24000
Receiver Gain	30
Relaxation Delay	1.0000
Pulse Width	4.0000
Acquisition Time	1.3107
Acquisition Date	2024-06-06T17:29:57
Modification Date	2024-06-07T08:55:04
Spectrum Quality	0.268
Spectrometer Frequency	100.53
Spectral Width	25000.0
Lowest Frequency	-1439.4
Nucleus	13C
Acquired Size	32768
Spectral Size	65536
Digital Resolution	0.38

^{13}C NMR spectrum (100 MHz, $CDCl_3$) of compound 1b

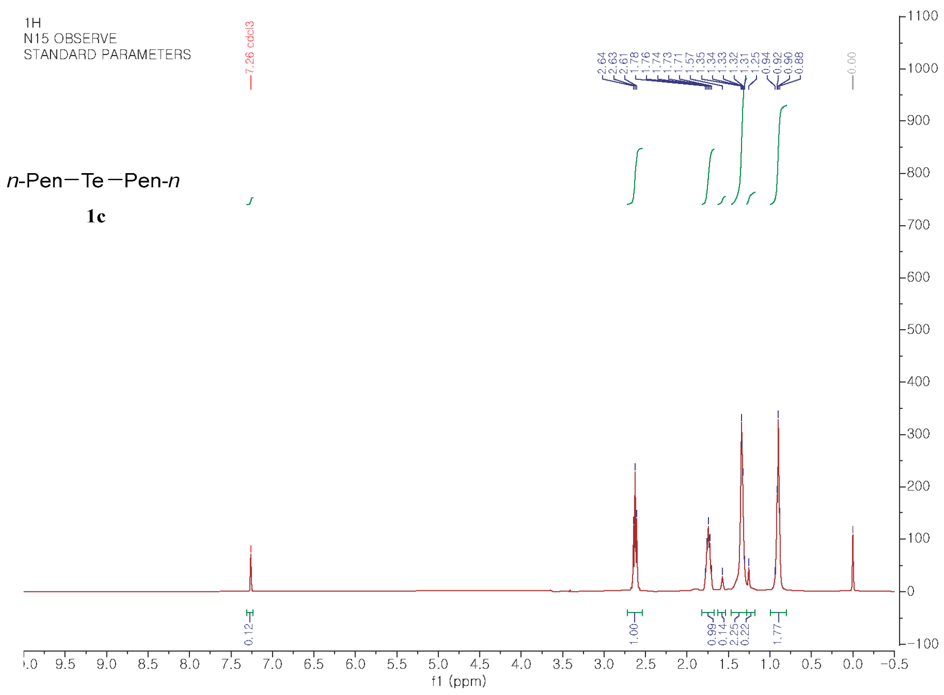

Parameter	Value
Data File Name	F:/ NMR/ 240311-KCR-687-A/ 1H/ fid
Title	1H
Instrument	vnmrs
Solvent	cdcl3
Temperature	30.0
Pulse Sequence	s2pul
Experiment	1D
Probe	5mm_ONE_probe
Number of Scans	16
Receiver Gain	40
Relaxation Delay	1.0000
Pulse Width	3.5500
Acquisition Time	2.5559
Acquisition Date	2024-03-11T16:50:13
Modification Date	2024-03-11T16:51:00
Spectrum Quality	0.000
Spectrometer Frequency	399.75
Spectral Width	6410.3
Lowest Frequency	-806.6
Nucleus	1H
Acquired Size	16384
Spectral Size	65536
Digital Resolution	0.10

^{1}H NMR spectrum (400 MHz, $CDCl_3$) of compound 1c

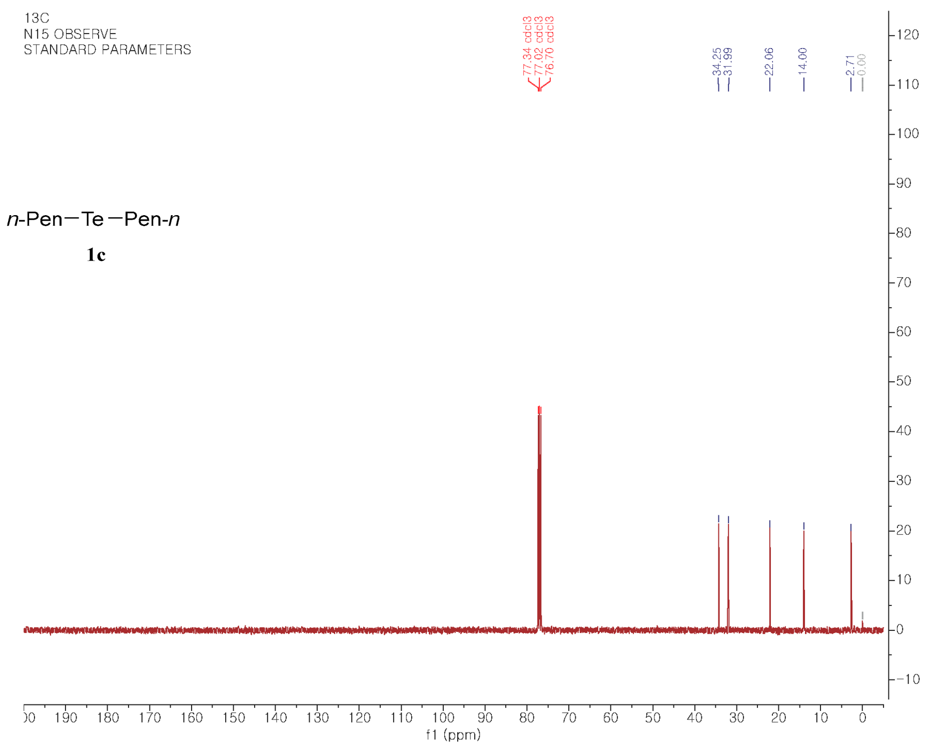

Parameter	Value
Data File Name	F:/ NMR/ 240311-KCR-687-A/ 13C/ fid
Title	13C
Instrument	vnmrs
Solvent	cdcl3
Temperature	30.0
Pulse Sequence	s2pul
Experiment	1D
Probe	5mm_ONE_probe
Number of Scans	960
Receiver Gain	30
Relaxation Delay	1.0000
Pulse Width	4.0000
Acquisition Time	1.3107
Acquisition Date	2024-03-11T17:02:41
Modification Date	2024-03-11T17:41:24
Spectrum Quality	0.000
Spectrometer Frequency	100.53
Spectral Width	25000.0
Lowest Frequency	-1443.2
Nucleus	13C
Acquired Size	32768
Spectral Size	65536
Digital Resolution	0.38

^{13}C NMR spectrum (100 MHz, $CDCl_3$) of compound 1c

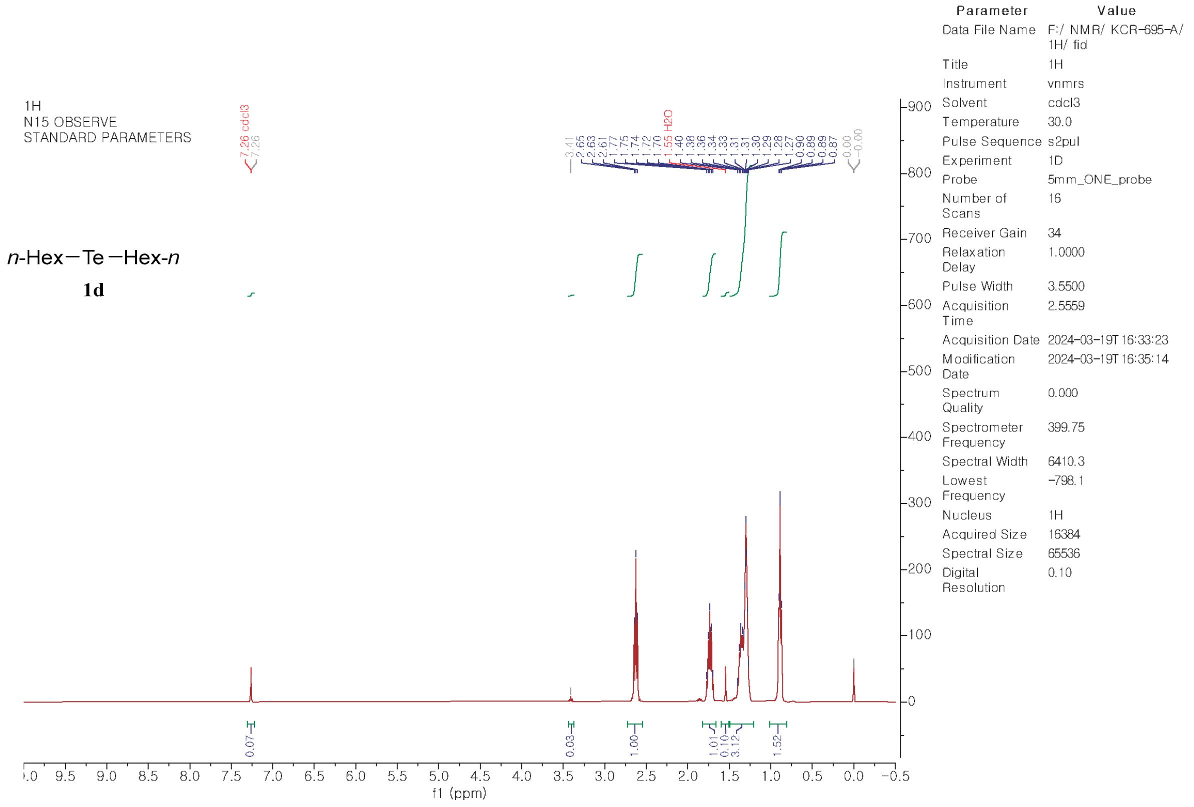

^{1}H NMR spectrum (400 MHz, $CDCl_3$) of compound 1d

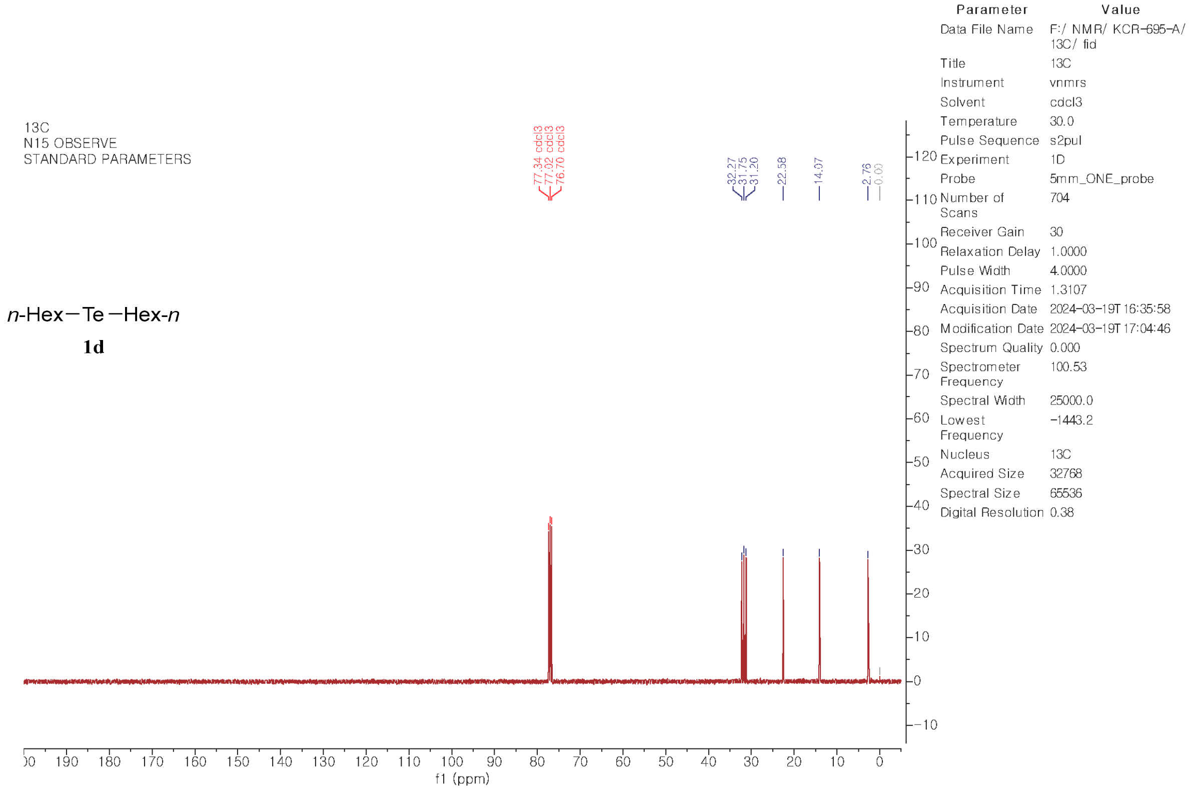

^{13}C NMR spectrum (100 MHz, $CDCl_3$) of compound 1d

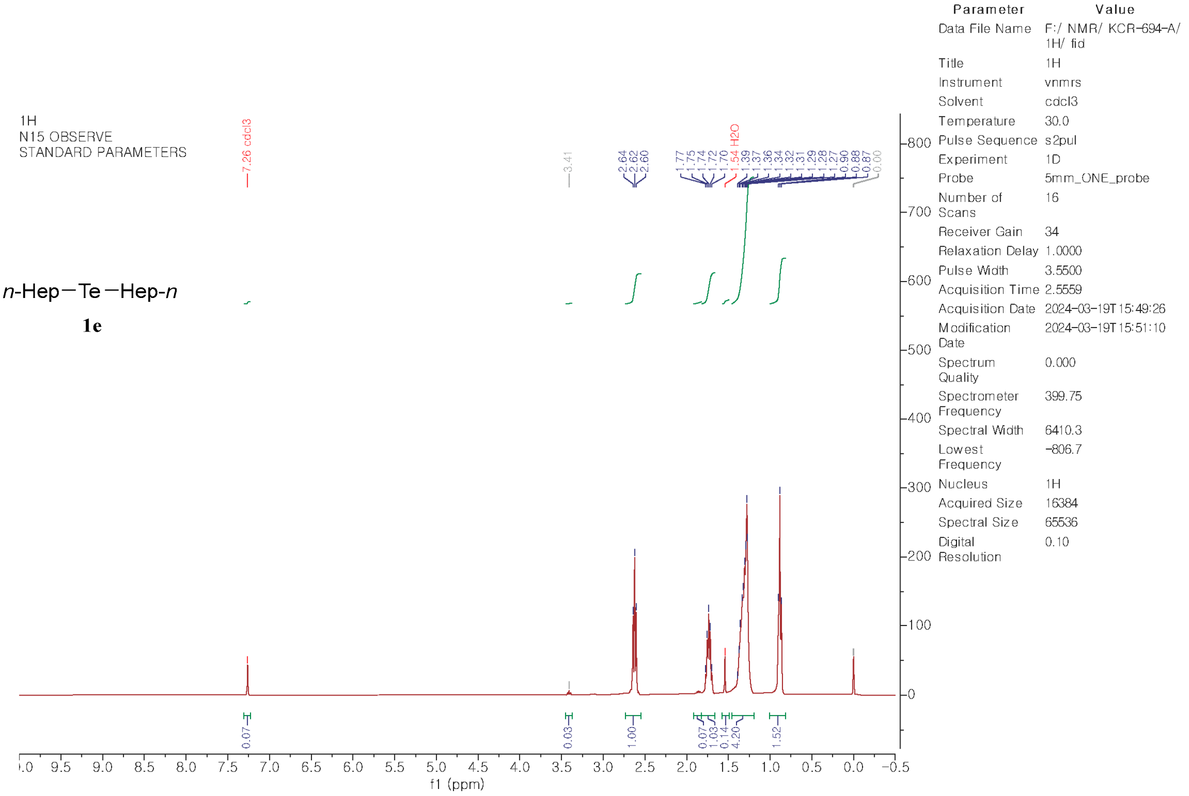

^{1}H NMR spectrum (400 MHz, $CDCl_3$) of compound 1e

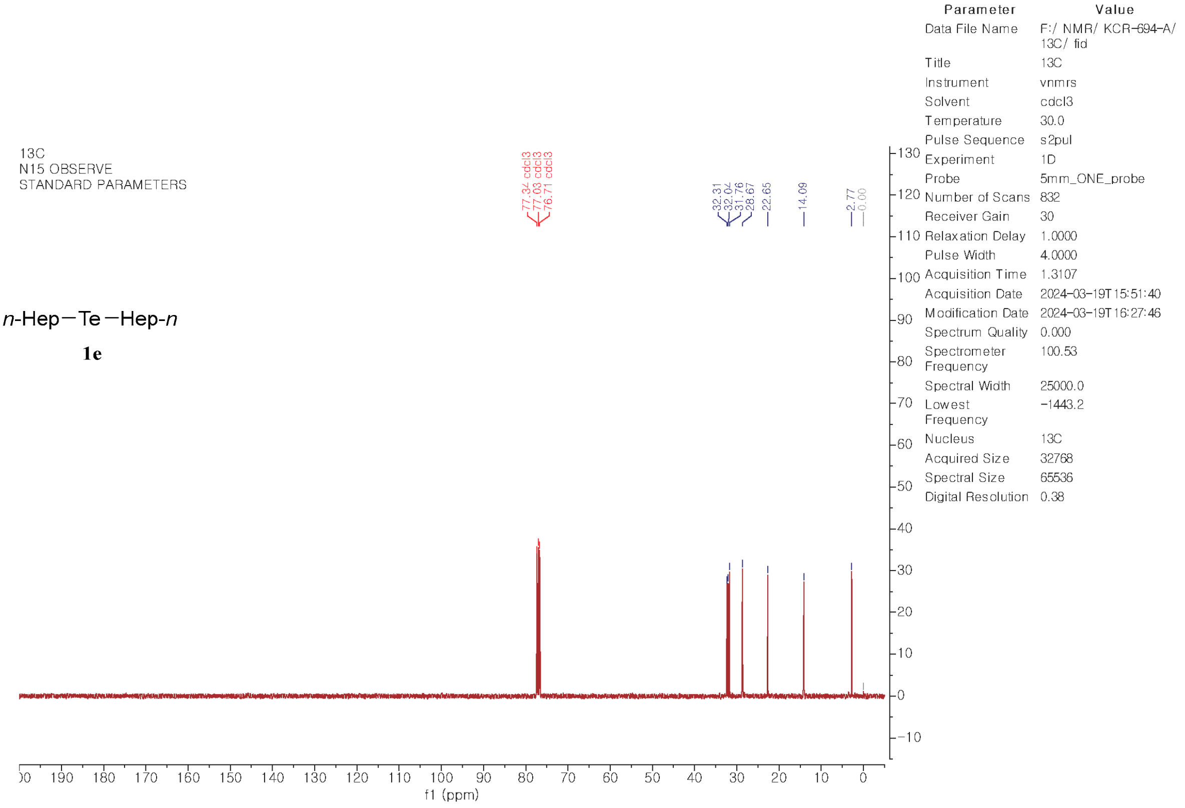

^{13}C NMR spectrum (100 MHz, $CDCl_3$) of compound 1e

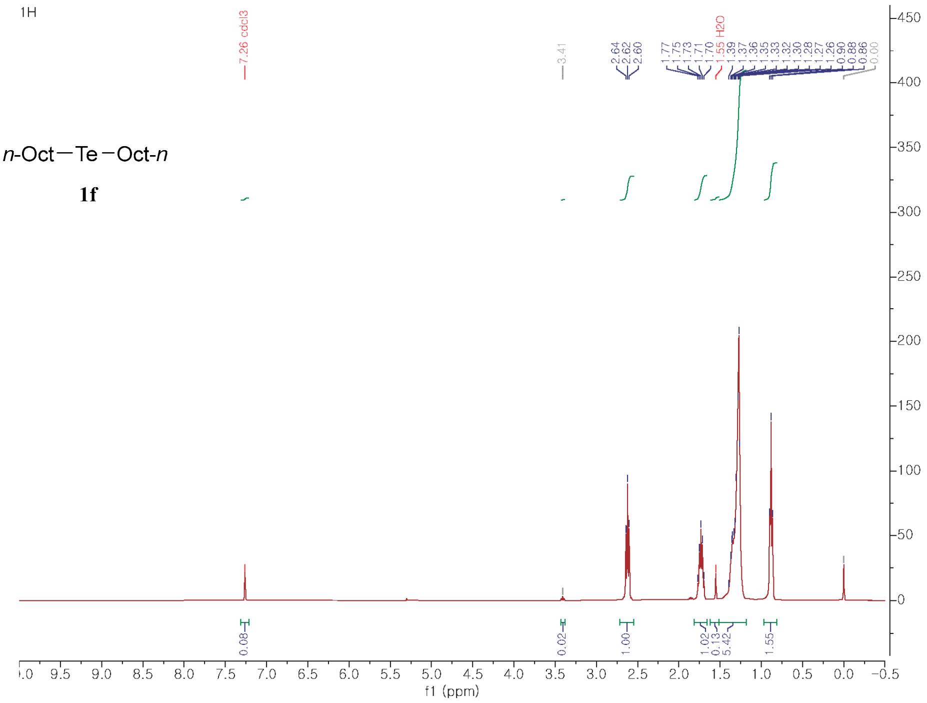

Parameter	Value
Data File Name	F:/ NMR/ KCR-697-A/ 1H/ fid
Title	1H
Instrument	vnmrs
Solvent	cdcl3
Temperature	30.0
Pulse Sequence	s2pul
Experiment	1D
Probe	5mm ONE probe
Number of Scans	8
Receiver Gain	34
Relaxation Delay	1.0000
Pulse Width	3.5500
Acquisition Time	2.5559
Acquisition Date	2024-03-21T18:55:42
Modification Date	2024-03-21T18:56:16
Spectrum Quality	0.000
Spectrometer Frequency	399.75
Spectral Width	6410.3
Lowest Frequency	-806.6
Nucleus	1H
Acquired Size	16384
Spectral Size	65536
Digital Resolution	0.10

^{1}H NMR spectrum (400 MHz, $CDCl_3$) of compound 1f

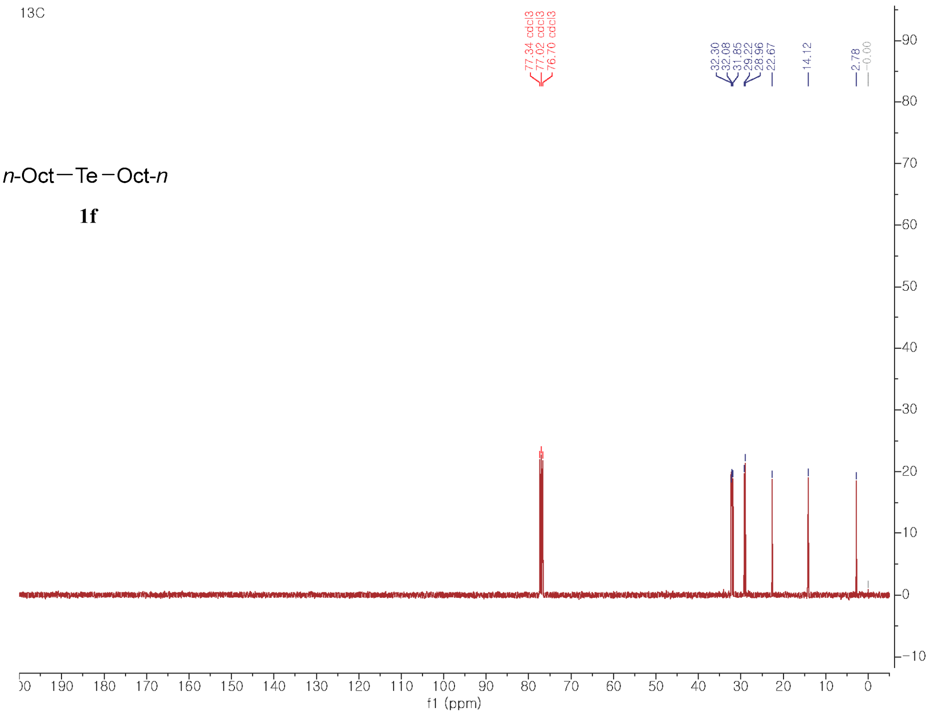

Parameter	Value
Data File Name	F:/ NMR/ KCR-697-A/ 13C/ fid
Title	13C
Instrument	vnmrs
Solvent	cdcl3
Temperature	30.0
Pulse Sequence	s2pul
Experiment	1D
Probe	5mm_ONE_probe
Number of Scans	576
Receiver Gain	30
Relaxation Delay	1.0000
Pulse Width	4.0000
Acquisition Time	1.3107
Acquisition Date	2024-03-21T18:58:15
Modification Date	2024-03-21T19:22:14
Spectrum Quality	0.000
Spectrometer Frequency	100.53
Spectral Width	25000.0
Lowest Frequency	-1443.2
Nucleus	13C
Acquired Size	32768
Spectral Size	65536
Digital Resolution	0.38

^{13}C NMR spectrum (100 MHz, $CDCl_3$) of compound 1f

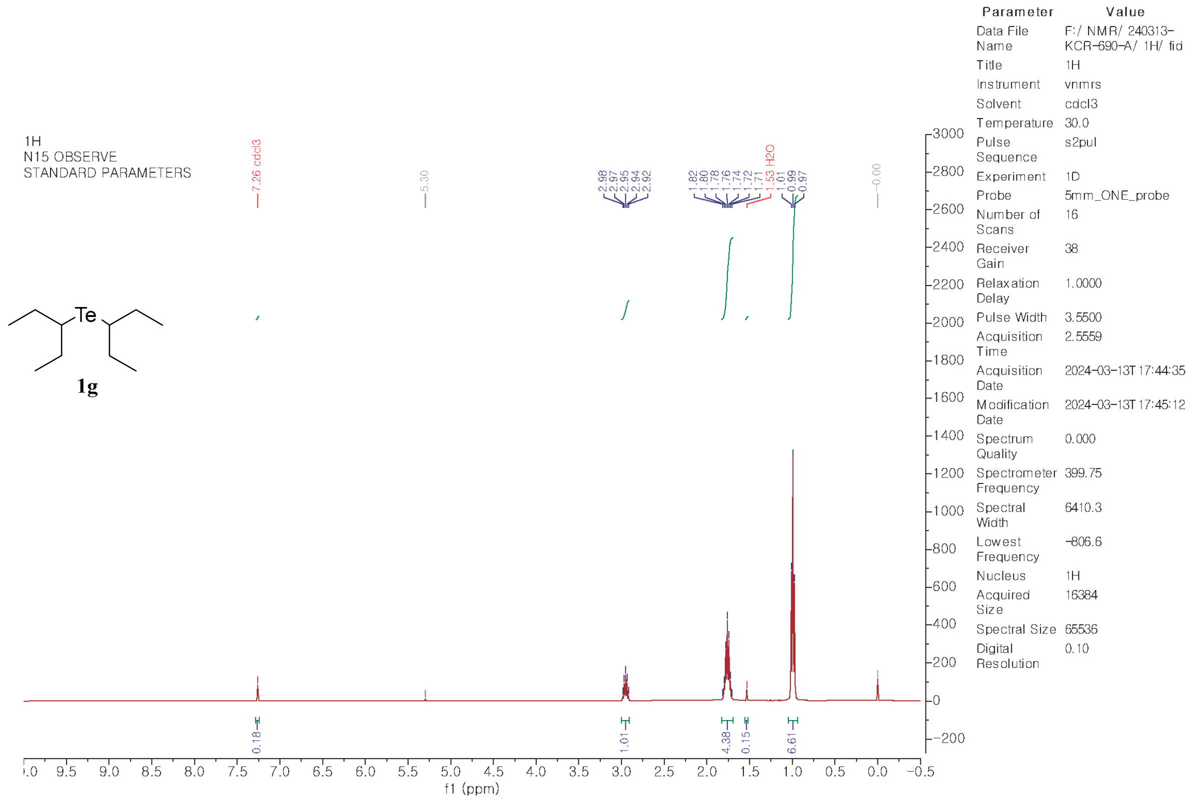

^{1}H NMR spectrum (400 MHz, $CDCl_3$) of compound 1g

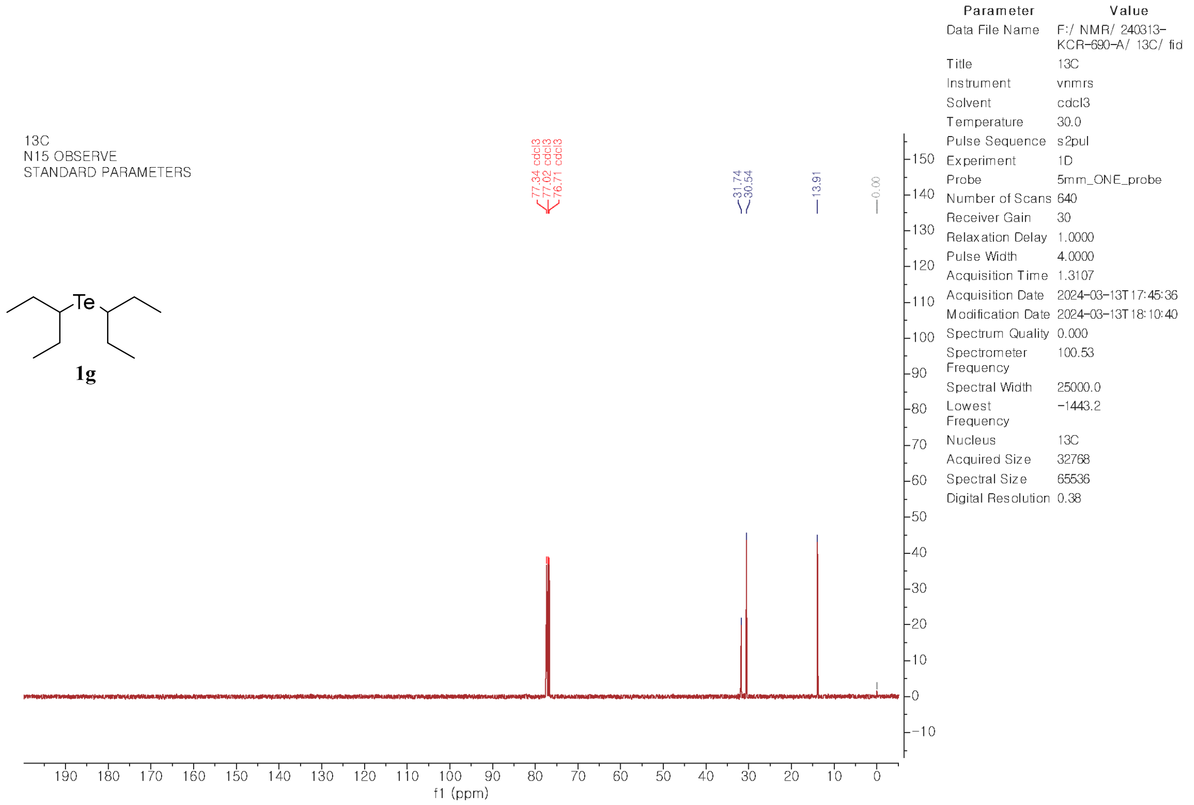

^{13}C NMR spectrum (100 MHz, $CDCl_3$) of compound 1g

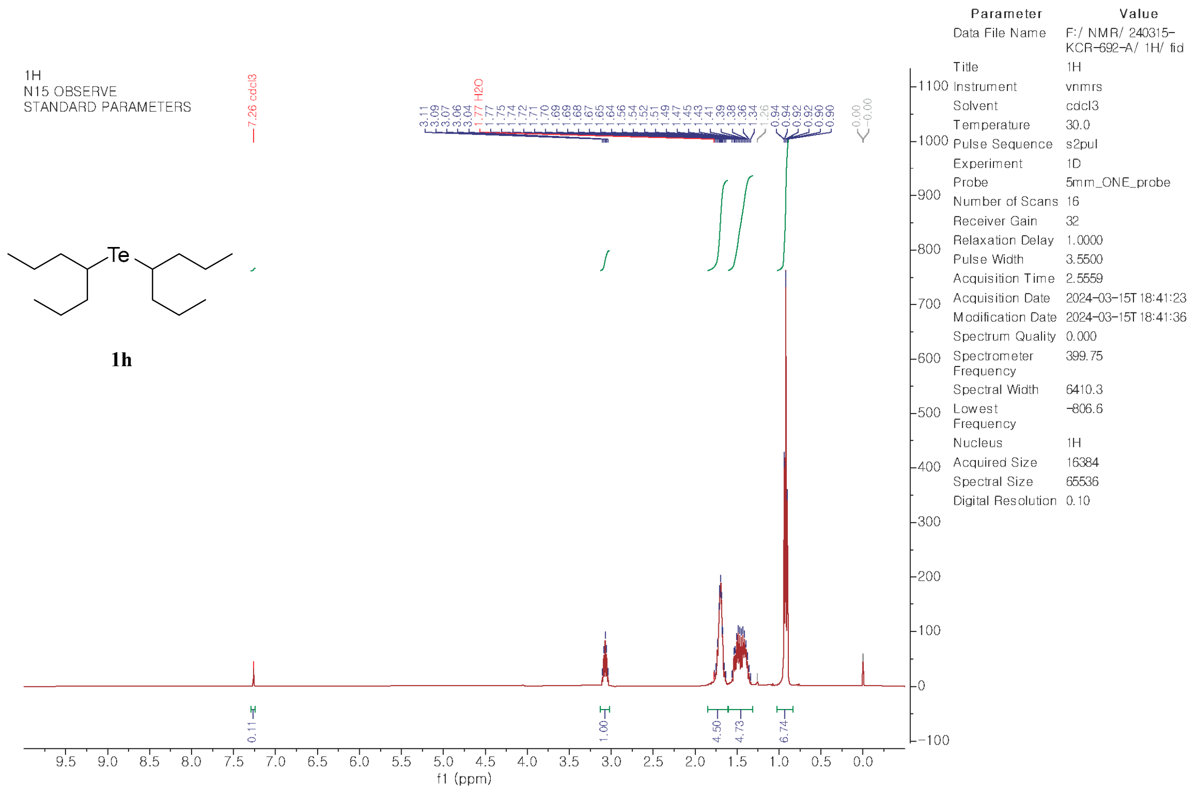

^{1}H NMR spectrum (400 MHz, $CDCl_3$) of compound 1h

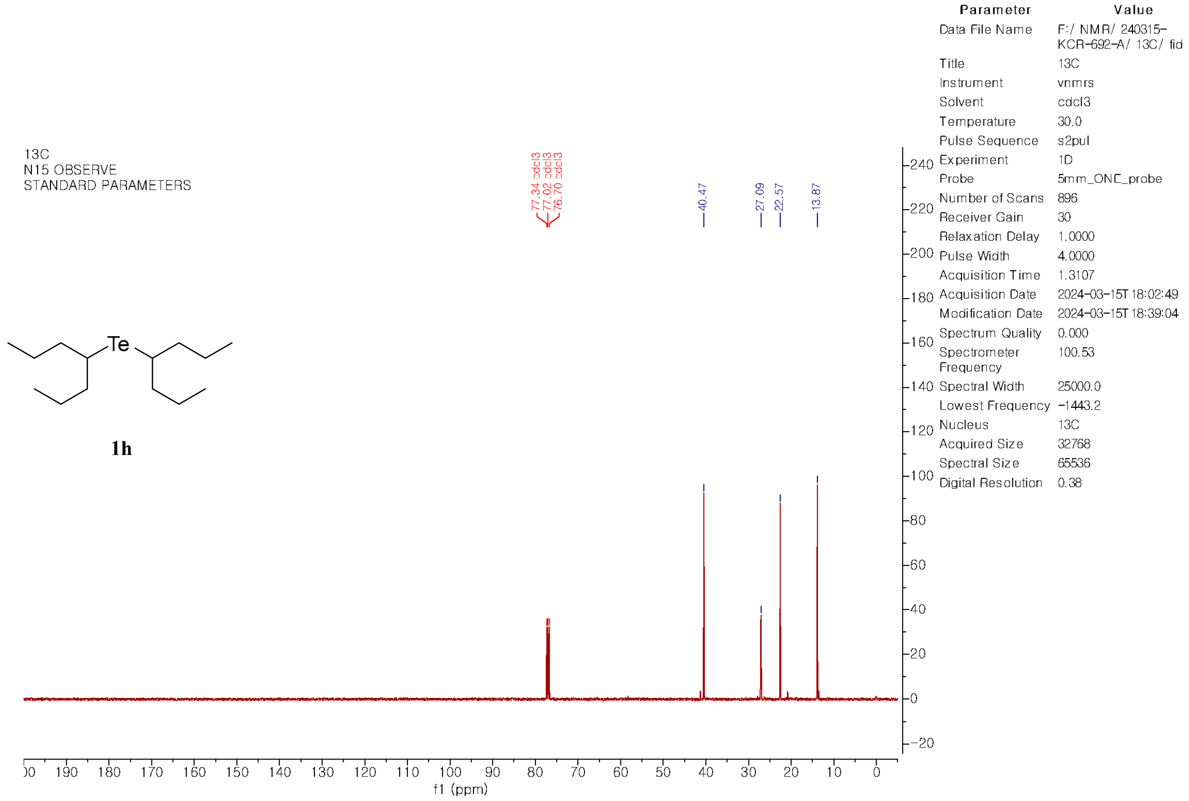

^{13}C NMR spectrum (100 MHz, $CDCl_3$) of compound 1h

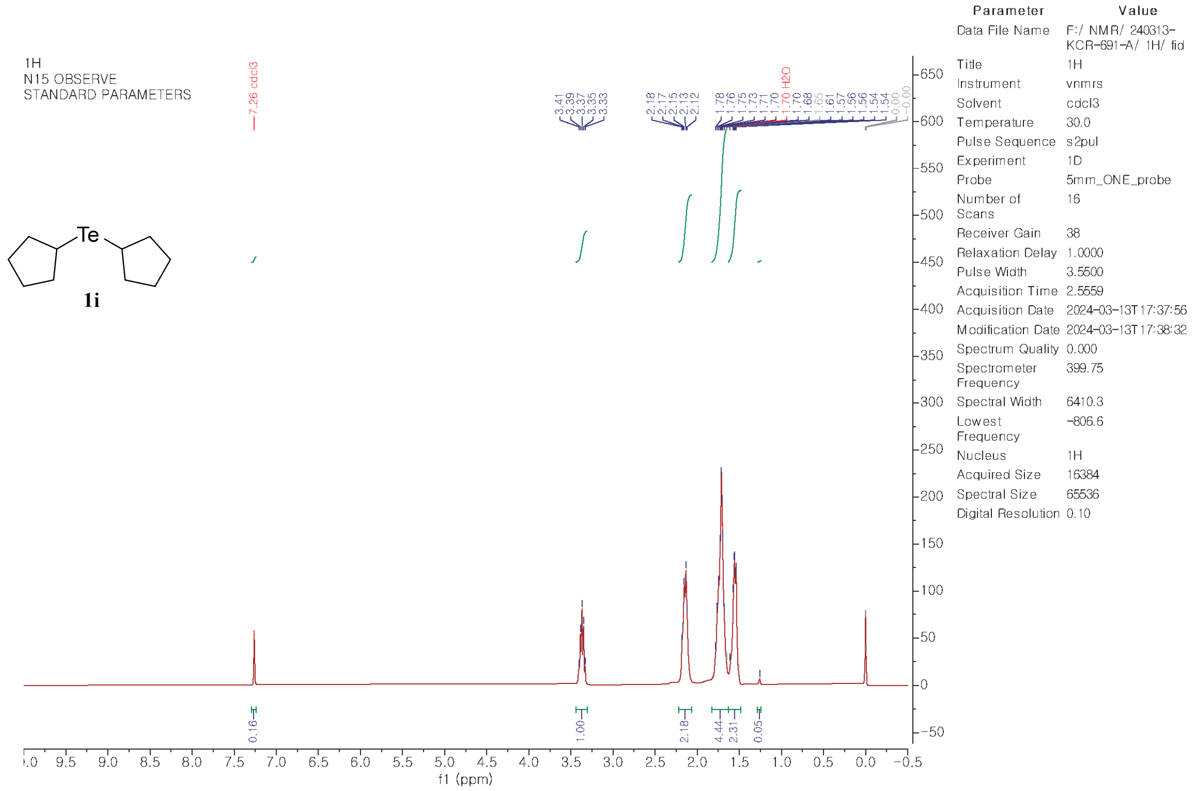

^{1}H NMR spectrum (400 MHz, $CDCl_3$) of compound 1i

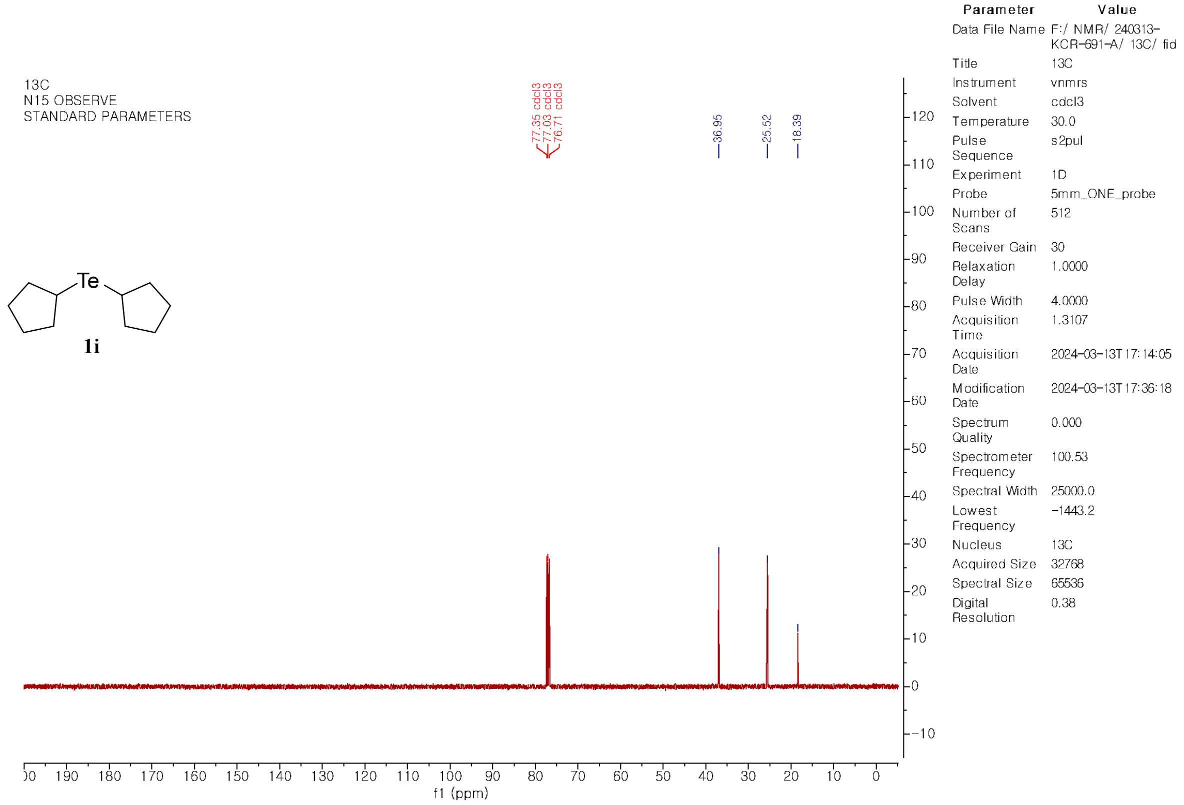

^{13}C NMR spectrum (100 MHz, $CDCl_3$) of compound 1i

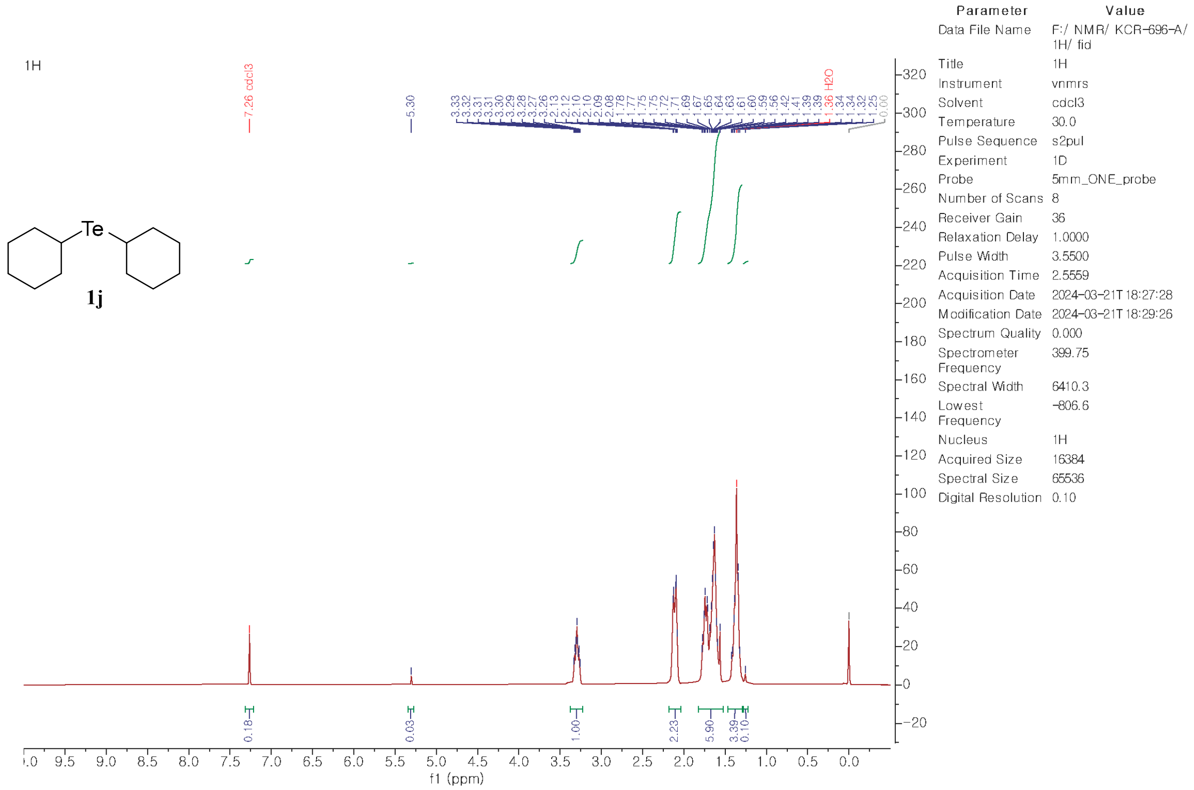

^{1}H NMR spectrum (400 MHz, $CDCl_3$) of compound 1j

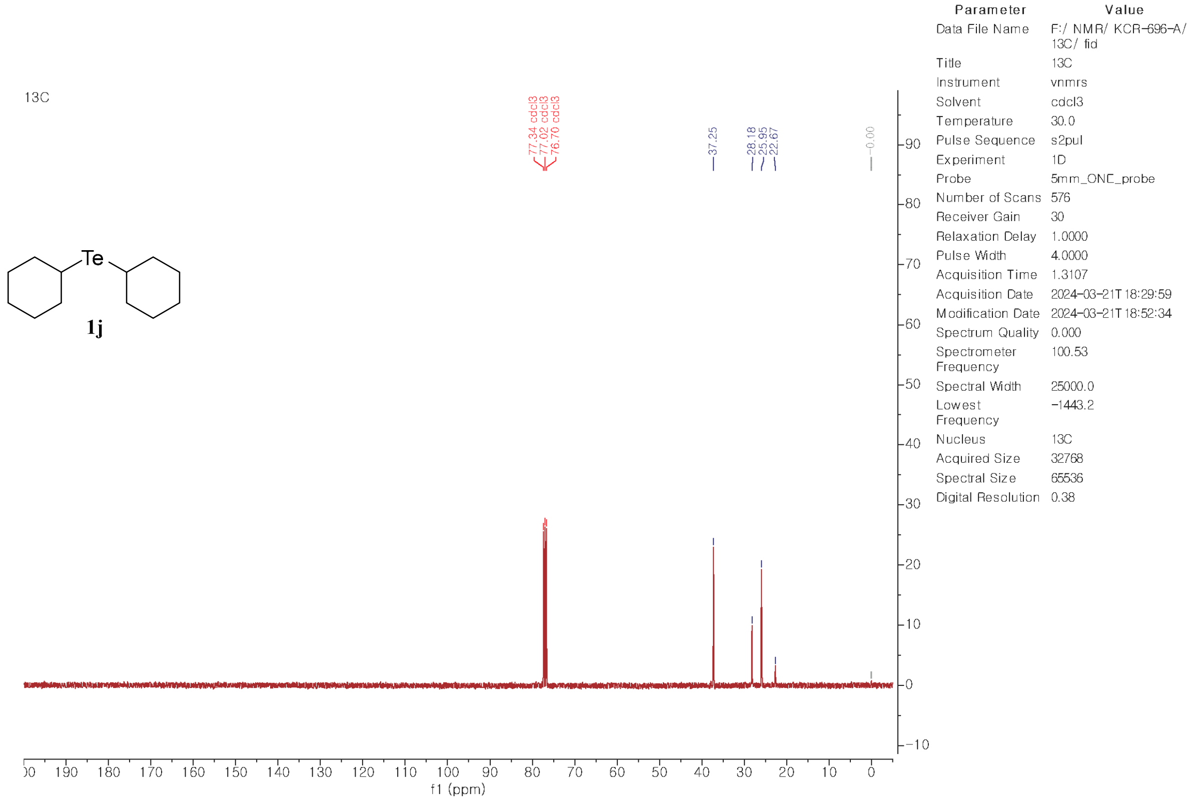

^{13}C NMR spectrum (100 MHz, $CDCl_3$) of compound 1j

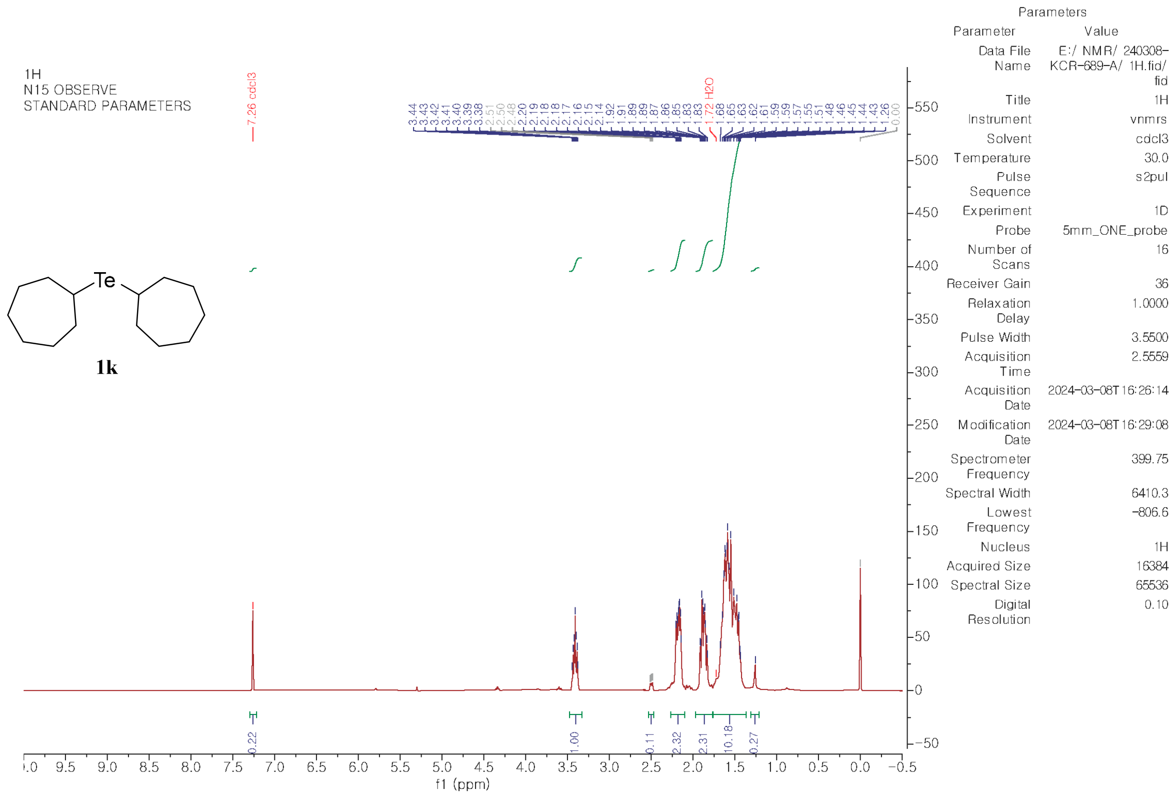

^{1}H NMR spectrum (400 MHz, $CDCl_3$) of compound 1k

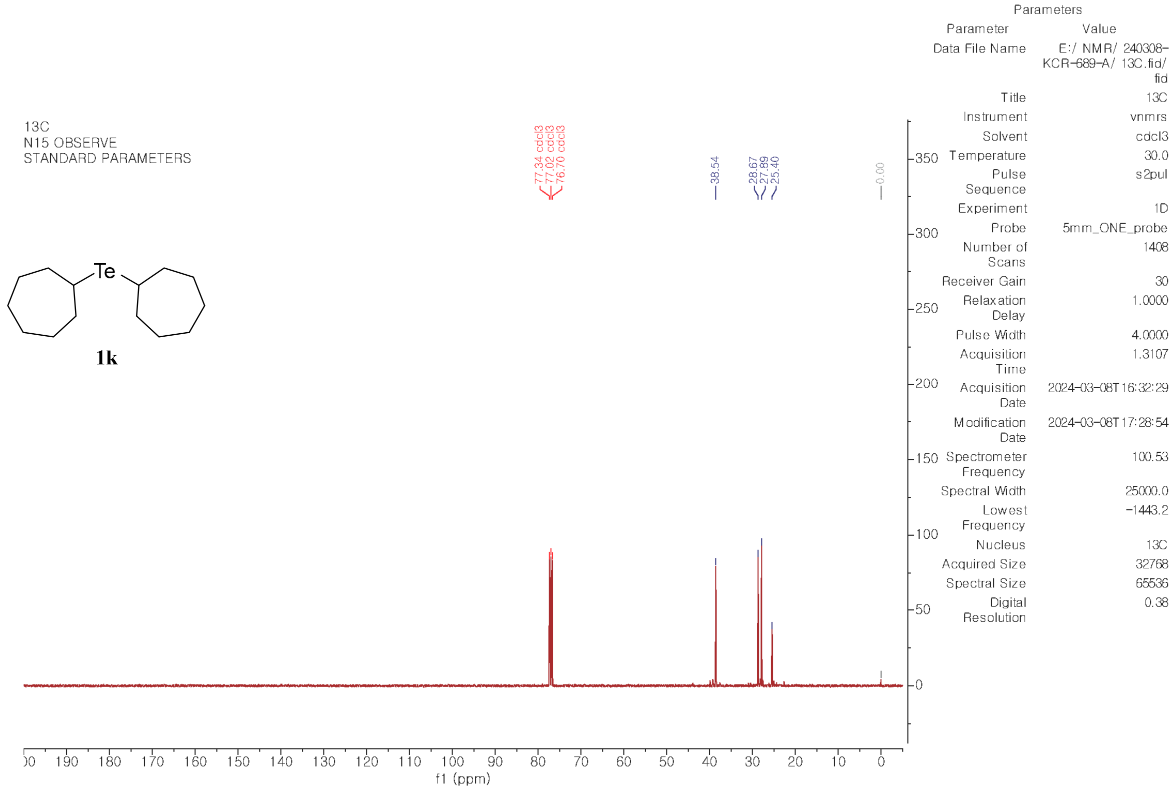

^{13}C NMR spectrum (100 MHz, $CDCl_3$) of compound 1k

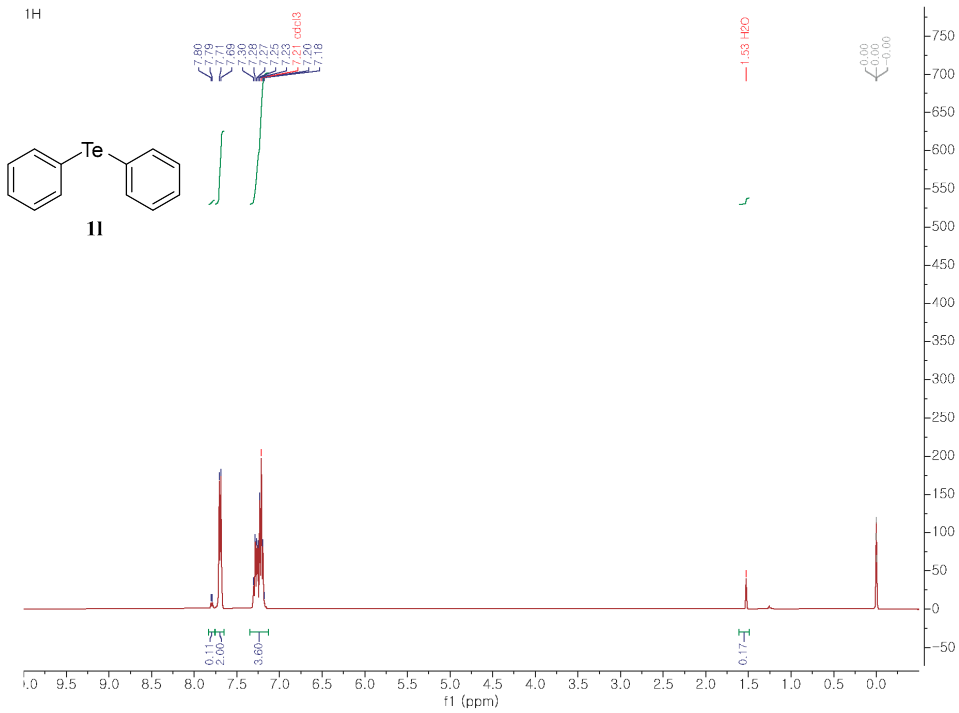

Parameter	Value
Data File Name	F:/ NMR/ KCR-710-A+B/ 1H/ fid
Title	1H
Instrument	vnmrs
Solvent	cdcl3
Temperature	30.0
Pulse Sequence	s2pul
Experiment	1D
Probe	5mm_ONE_probe
Number of Scans	8
Receiver Gain	44
Relaxation Delay	1.0000
Pulse Width	3.5500
Acquisition Time	2.5559
Acquisition Date	2024-04-18T15:43:39
Modification Date	2024-04-18T15:43:54
Spectrum Quality	0.000
Spectrometer Frequency	399.75
Spectral Width	6410.3
Lowest Frequency	-806.6
Nucleus	1H
Acquired Size	16384
Spectral Size	65536
Digital Resolution	0.10

^{1}H NMR spectrum (400 MHz, $CDCl_3$) of compound 1l

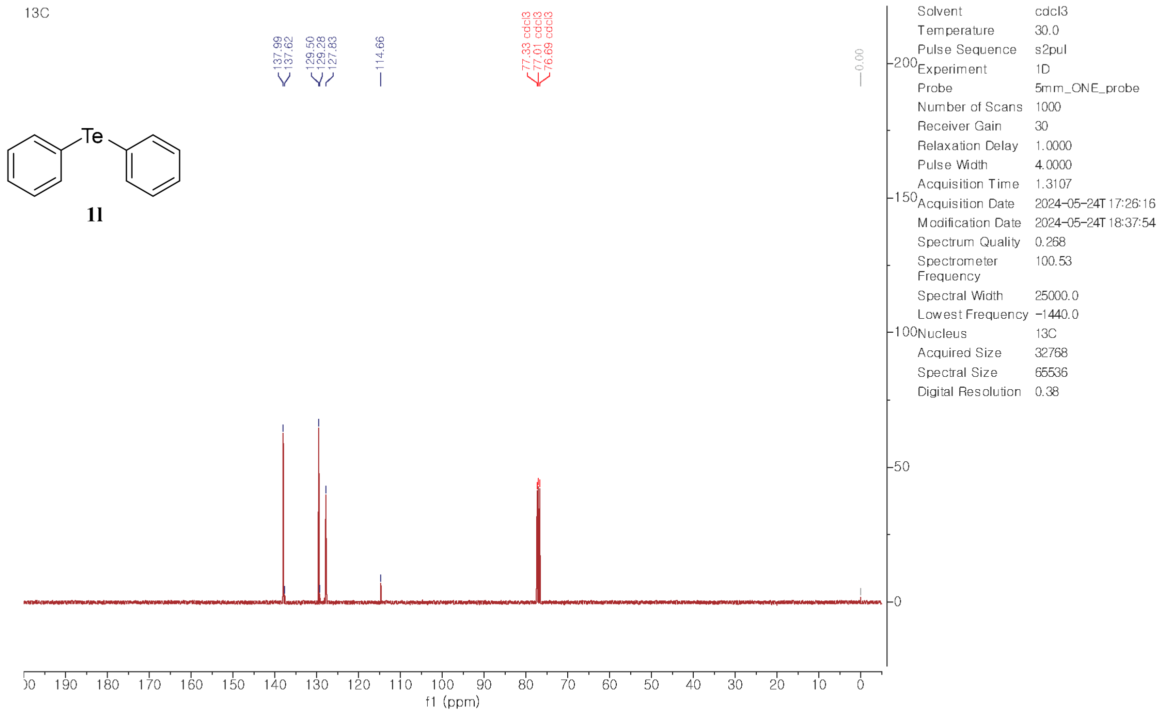

Parameter	Value
Data File Name	F:/ NMR/ KCR-710-A+X/ 13C/ fid
Title	13C
Instrument	vnmrs
Solvent	cdcl3
Temperature	30.0
Pulse Sequence	s2pul
Experiment	1D
Probe	5mm_ONE_probe
Number of Scans	1000
Receiver Gain	30
Relaxation Delay	1.0000
Pulse Width	4.0000
Acquisition Time	1.3107
Acquisition Date	2024-05-24T17:26:16
Modification Date	2024-05-24T18:37:54
Spectrum Quality	0.268
Spectrometer Frequency	100.53
Spectral Width	25000.0
Lowest Frequency	-1440.0
Nucleus	13C
Acquired Size	32768
Spectral Size	65536
Digital Resolution	0.38

^{13}C NMR spectrum (100 MHz, $CDCl_3$) of compound 1l

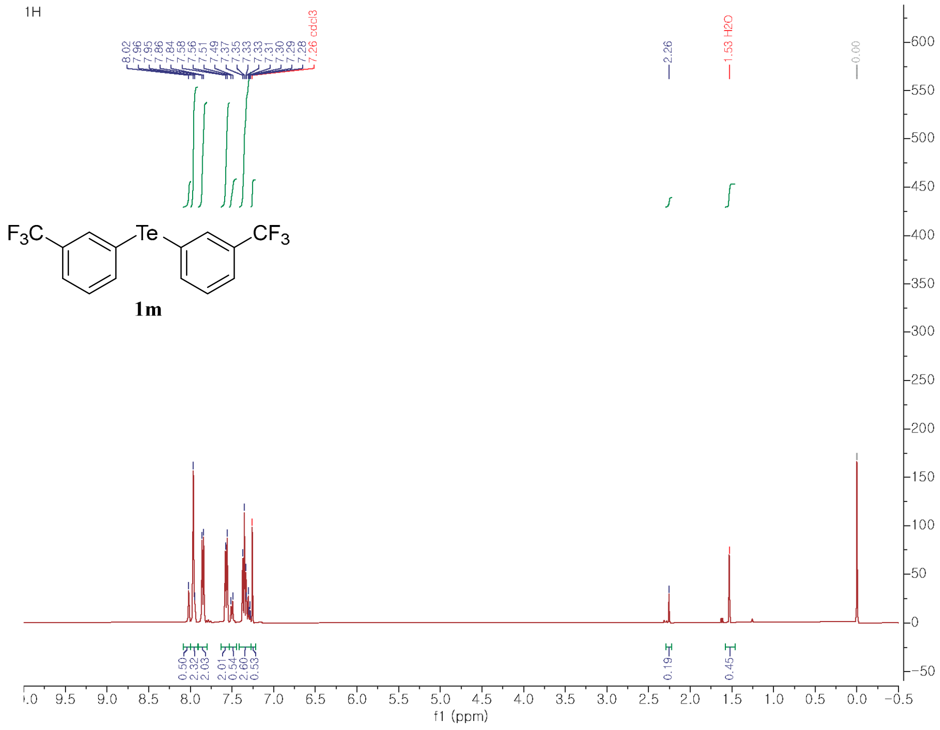

Parameter	Value
Data File Name	E:/ NMR/ KCR-735-A/ 1H/ fid
Title	1H
Comment	
Origin	Varian
Owner	
Site	
Instrument	vnmrs
Author	
Solvent	cdcl3
Temperature	30.0
Pulse Sequence	s2pul
Experiment	1D
Probe	5mm_ONE_probe
Number of Scans	8
Receiver Gain	44
Relaxation Delay	1.0000
Pulse Width	3.5500
Presaturation Frequency	
Acquisition Time	2.5559
Acquisition Date	2024-05-29T18:01:51
Modification Date	2024-05-29T18:02:00
Class	
Spectrometer Frequency	399.75
Spectral Width	6410.3
Lowest Frequency	-800.3
Nucleus	1H
Acquired Size	16384
Spectral Size	65536
Digital Resolution	0.10

^{1}H NMR spectrum (400 MHz, $CDCl_3$) of compound 1m

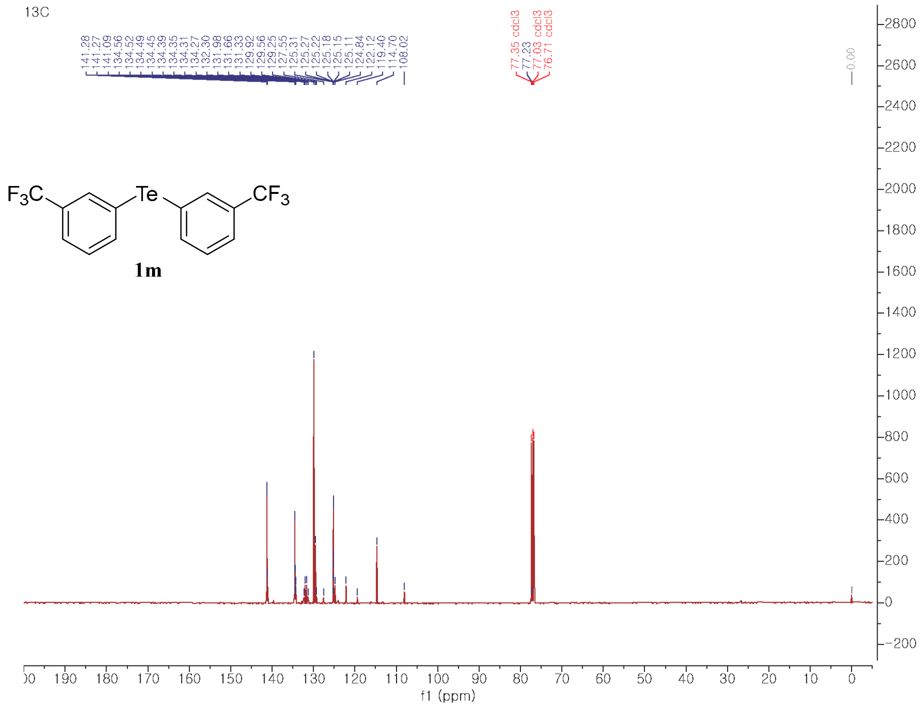

Parameter	Value
Data File Name	F:/ NMR/ 240610-KCR-735-A/ 13C/ fid
Title	13C
Instrument	vnmrs
Solvent	cdcl3
Temperature	30.0
Pulse Sequence	s2pul
Experiment	1D
Probe	5mm_ONE_probe
Number of Scans	20800
Receiver Gain	30
Relaxation Delay	1.0000
Pulse Width	4.0000
Acquisition Time	1.3107
Acquisition Date	2024-06-10T20:05:06
Modification Date	2024-06-11T09:28:36
Spectrum Quality	0.000
Spectrometer Frequency	100.53
Spectral Width	25000.0
Lowest Frequency	-1443.2
Nucleus	13C
Acquired Size	32768
Spectral Size	65536
Digital Resolution	0.38

^{13}C NMR spectrum (100 MHz, $CDCl_3$) of compound 1m

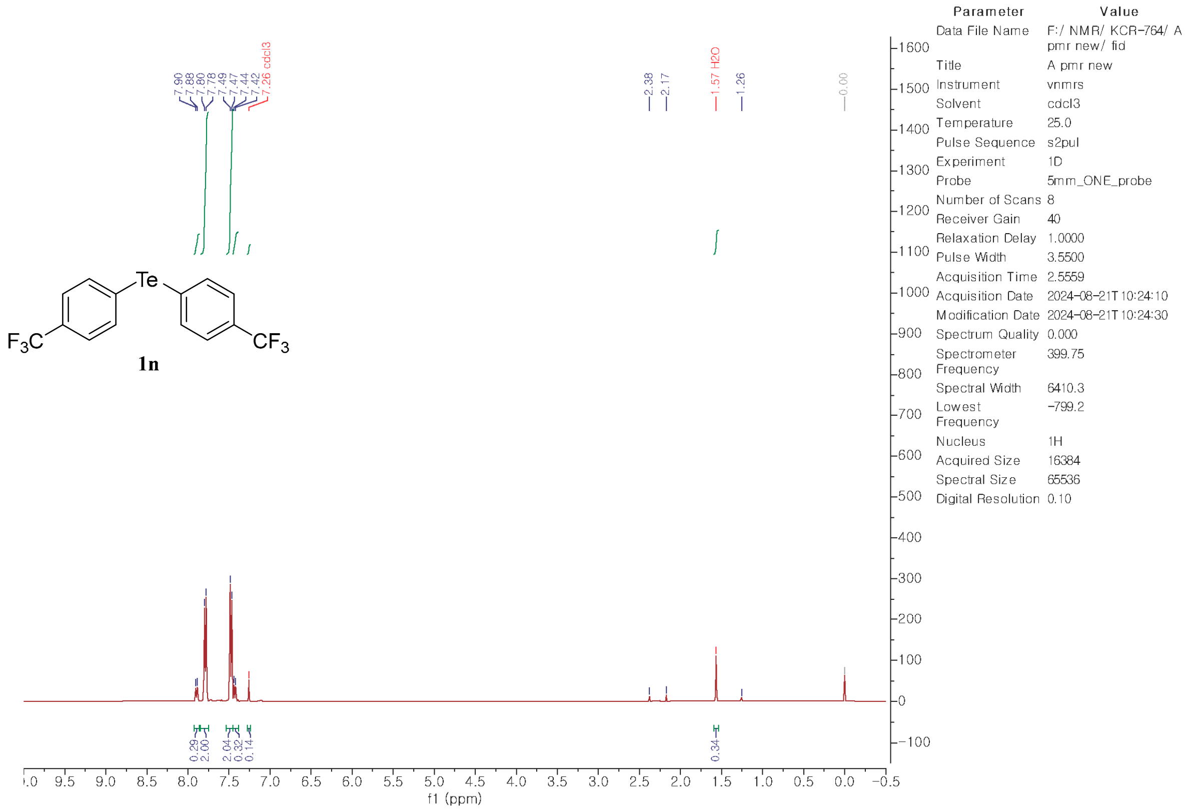

^{1}H NMR spectrum (400 MHz, CDCl$_3$) of compound 1n

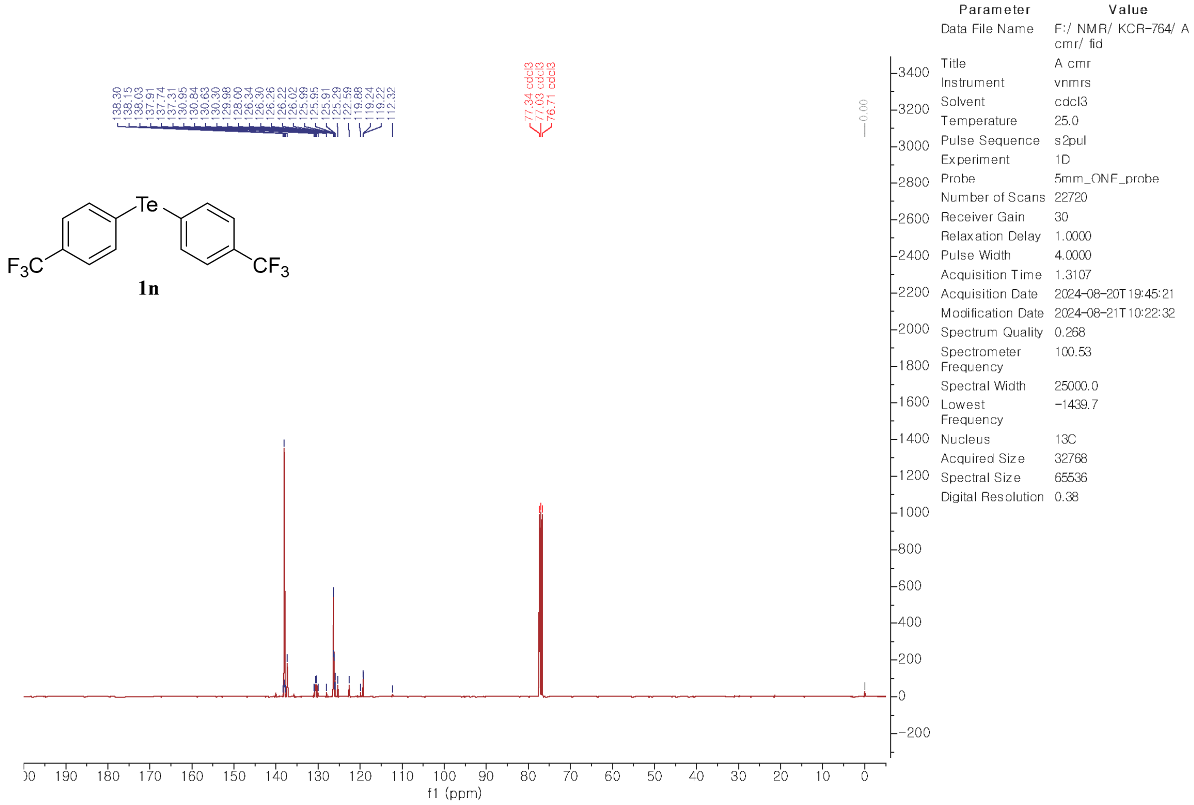

^{13}C NMR spectrum (100 MHz, CDCl$_3$) of compound 1n

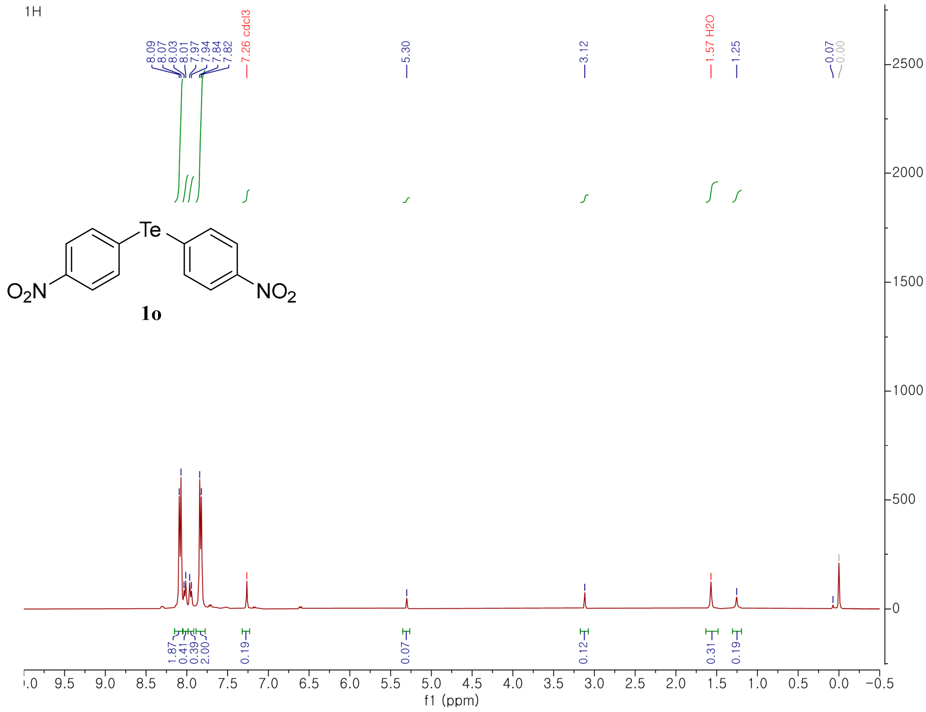

Parameter	Value
Data File Name	F:/ NMR/ KCR-716-A/ 1H/ fid
Title	1H
Instrument	vnmrs
Solvent	cdcl3
Temperature	30.0
Pulse Sequence	s2pul
Experiment	1D
Probe	5mm_ONE_probe
Number of Scans	32
Receiver Gain	42
Relaxation Delay	1.0000
Pulse Width	3.5500
Acquisition Time	2.5559
Acquisition Date	2024-06-11T15:42:05
Modification Date	2024-06-11T15:42:12
Spectrum Quality	-0.701
Spectrometer Frequency	399.75
Spectral Width	6410.3
Lowest Frequency	-797.1
Nucleus	1H
Acquired Size	16384
Spectral Size	65536
Digital Resolution	0.10

^{1}H NMR spectrum (400 MHz, $CDCl_3$) of compound 1o

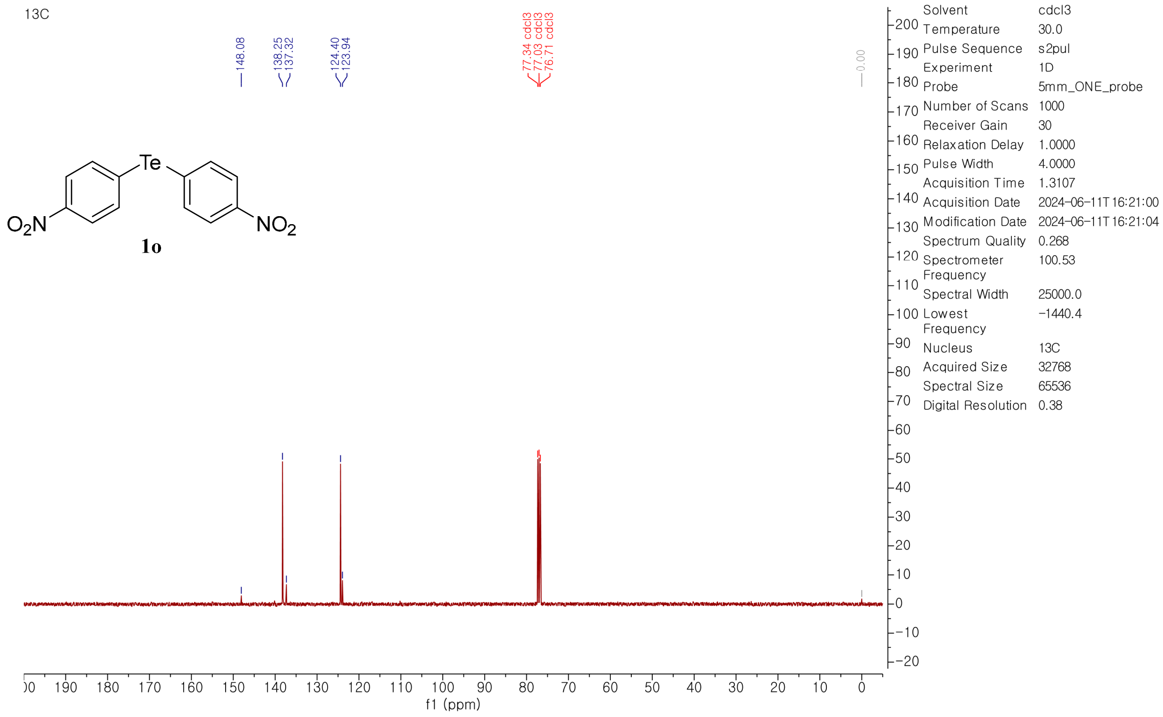

Parameter	Value
Data File Name	F:/ NMR/ KCR-716-A/ 13C/ fid
Title	13C
Instrument	vnmrs
Solvent	cdcl3
Temperature	30.0
Pulse Sequence	s2pul
Experiment	1D
Probe	5mm_ONE_probe
Number of Scans	1000
Receiver Gain	30
Relaxation Delay	1.0000
Pulse Width	4.0000
Acquisition Time	1.3107
Acquisition Date	2024-06-11T16:21:00
Modification Date	2024-06-11T16:21:04
Spectrum Quality	0.268
Spectrometer Frequency	100.53
Spectral Width	25000.0
Lowest Frequency	-1440.4
Nucleus	13C
Acquired Size	32768
Spectral Size	65536
Digital Resolution	0.38

^{13}C NMR spectrum (100 MHz, $CDCl_3$) of compound 1o

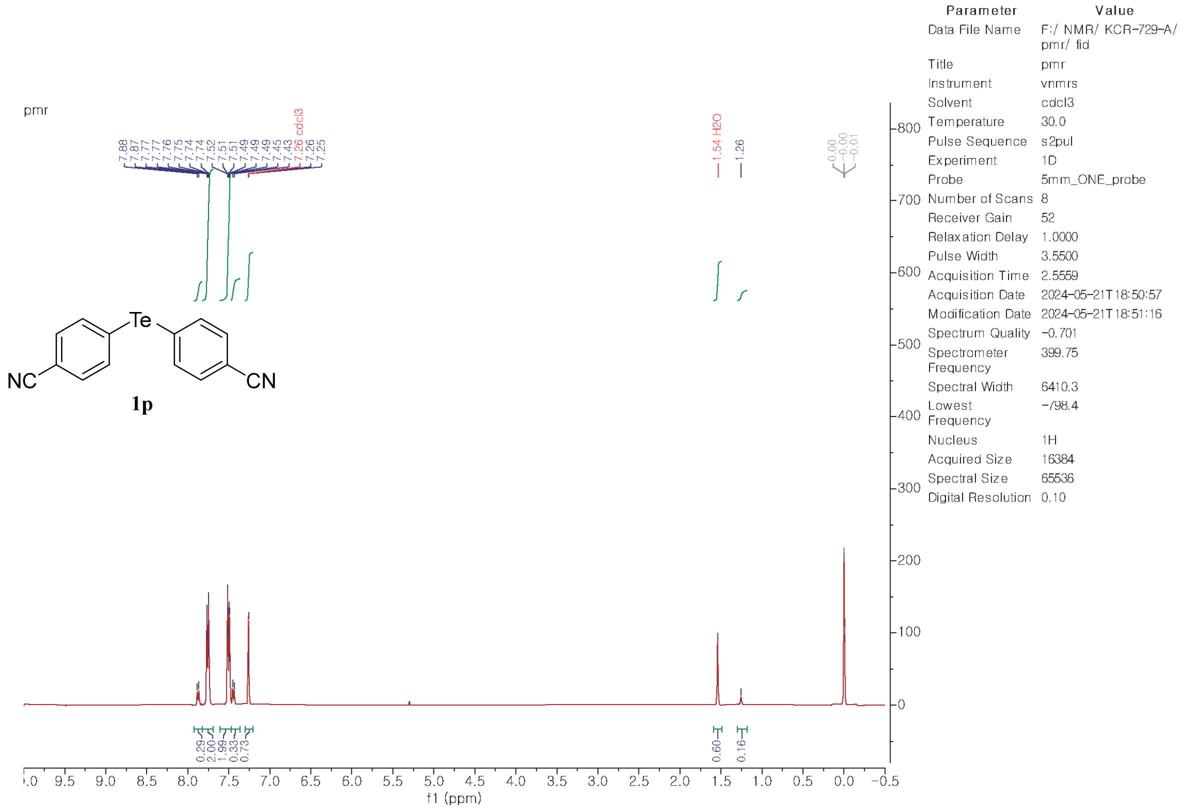

^{1}H NMR spectrum (400 MHz, $CDCl_3$) of compound 1p

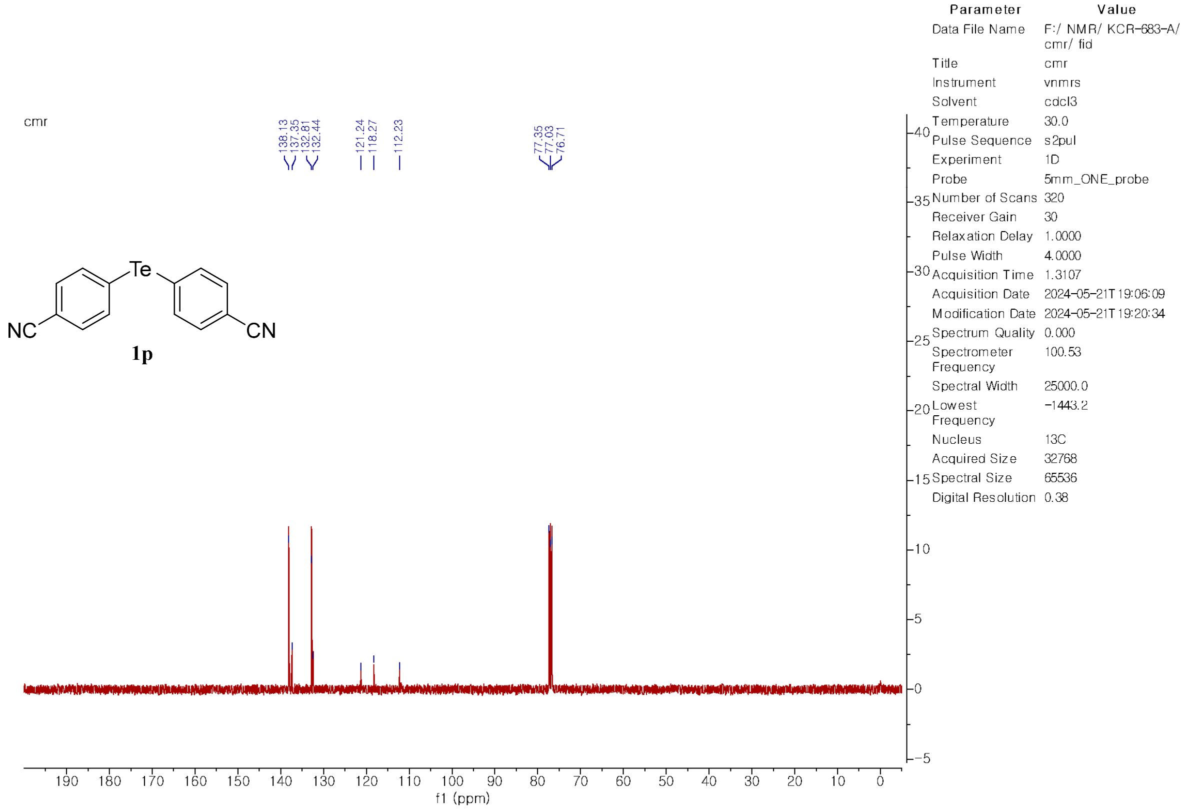

^{13}C NMR spectrum (100 MHz, $CDCl_3$) of compound 1p

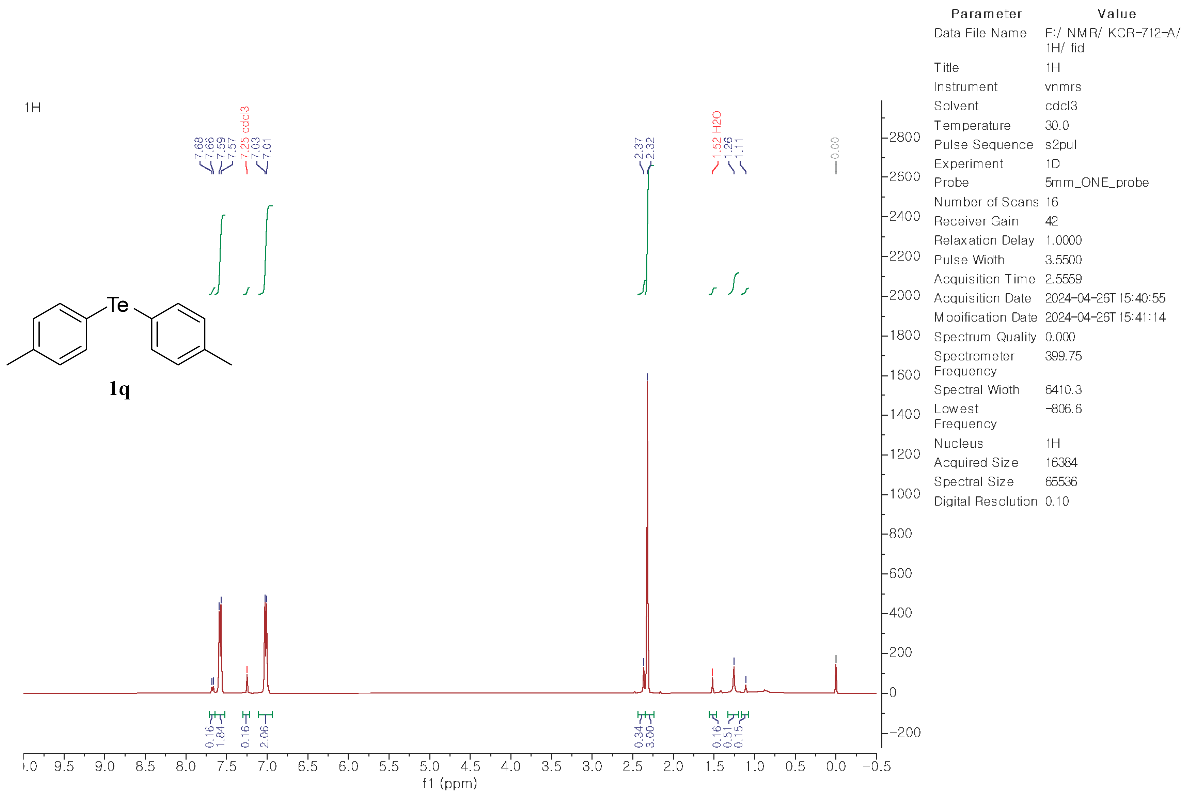

^{1}H NMR spectrum (400 MHz, $CDCl_3$) of compound 1q

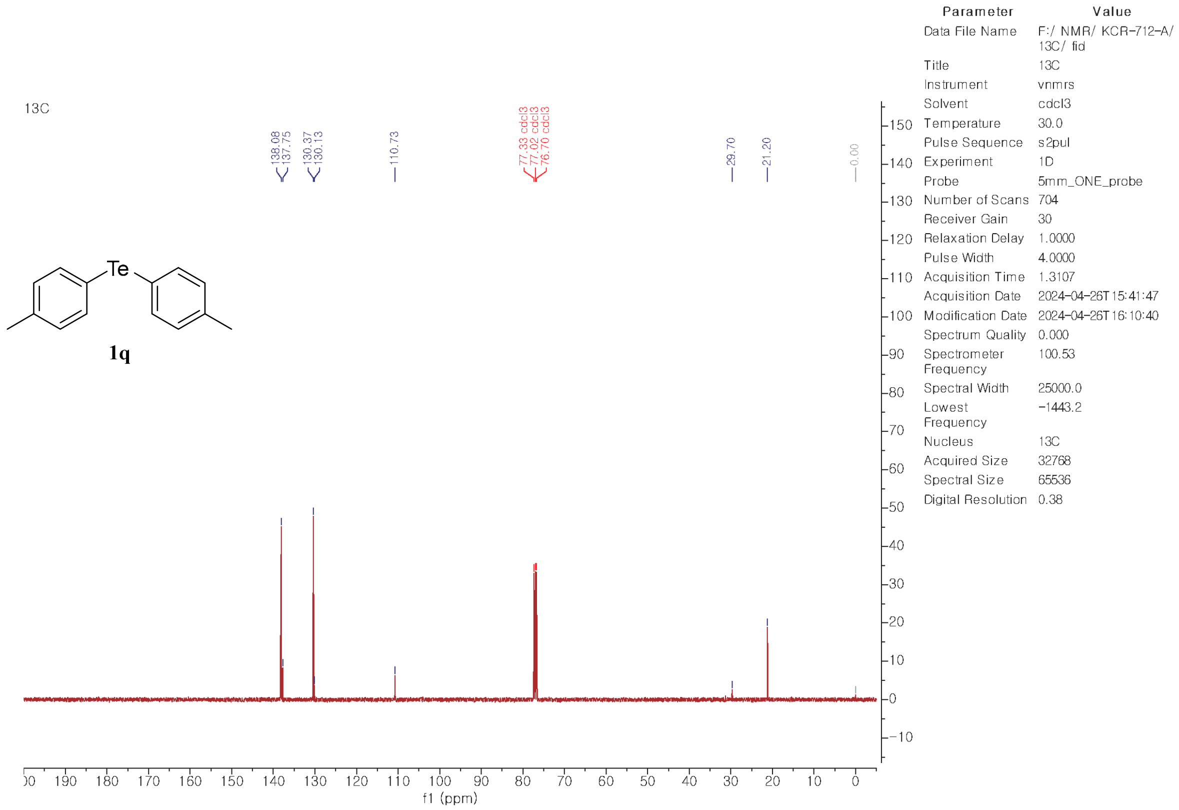

^{13}C NMR spectrum (100 MHz, $CDCl_3$) of compound 1q

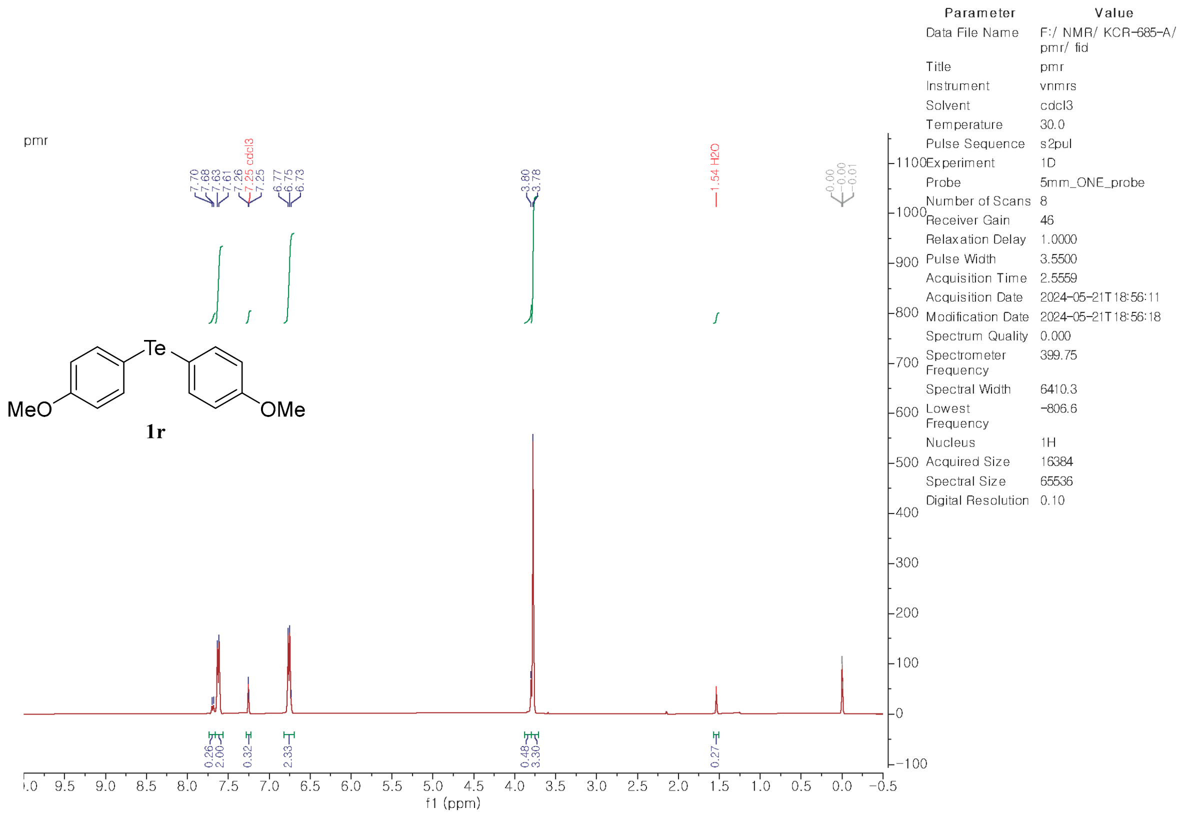

^{1}H NMR spectrum (400 MHz, $CDCl_3$) of compound 1r

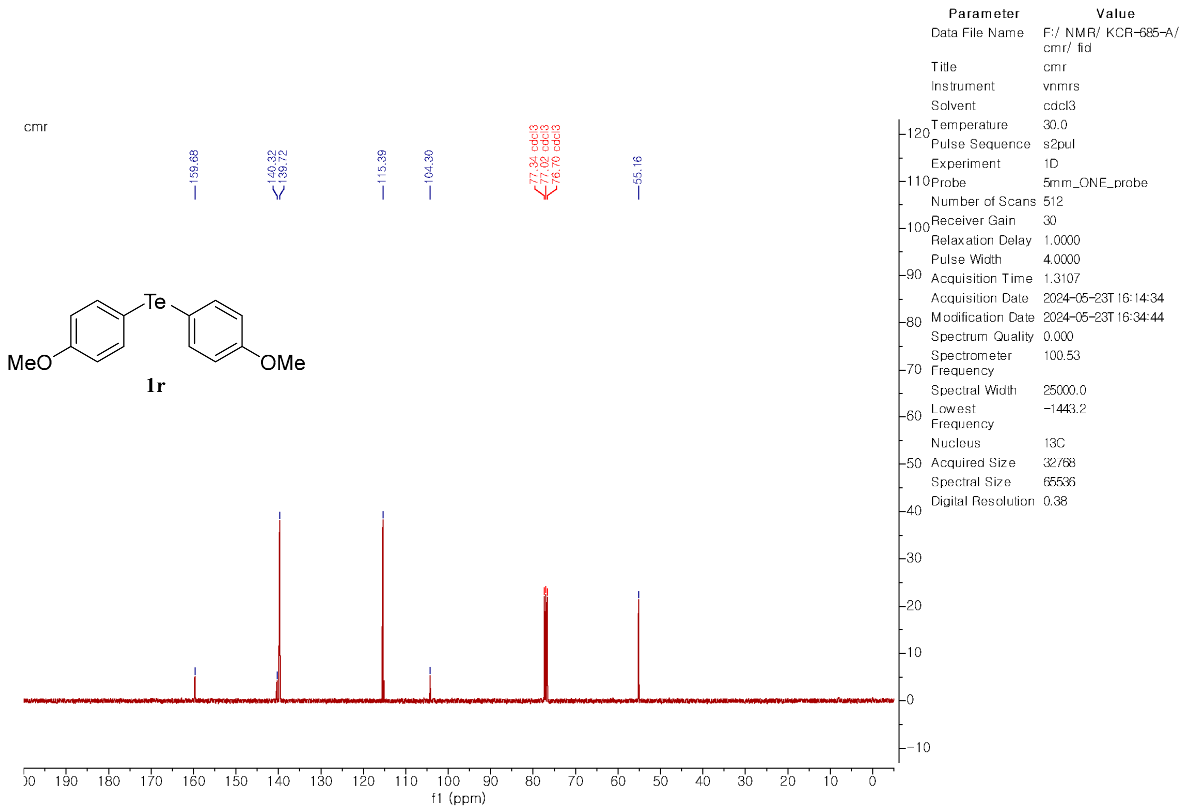

^{13}C NMR spectrum (100 MHz, $CDCl_3$) of compound 1r
